# Reversible Parahydrogen Induced Hyperpolarization of ^15^N in Unmodified Amino Acids Unraveled at High Magnetic Field

**DOI:** 10.1002/advs.202207112

**Published:** 2023-05-21

**Authors:** Ewoud Vaneeckhaute, Jean‐Max Tyburn, James G. Kempf, Johan A. Martens, Eric Breynaert

**Affiliations:** ^1^ COK‐kat Centre for Surface Chemistry and Catalysis—Characterization and Application Team KU Leuven Celestijnenlaan 200F, box 2461 Leuven B‐3001 Belgium; ^2^ NMRCoRe NMR/X‐Ray Platform for Convergence Research KU Leuven Celestijnenlaan 200F, box 2461 Leuven B‐3001 Belgium; ^3^ Univ Lyon CNRS, ENS Lyon UCBL Université de Lyon CRMN UMR 5280 Villeurbanne 69100 France; ^4^ Bruker Biospin 34 Rue de l'Industrie BP 10002 Wissembourg Cedex 67166 France; ^5^ Bruker Biospin 15 Fortune Dr. Billerica MA 01821 USA; ^6^ Deutsches Elektronen‐Synchrotron DESY – Centre for Molecular Water Science (CMWS) Notkestraße 85 22607 Hamburg Germany

**Keywords:** ^15^N‐NMR, amino acids, ammonia, hydrides, hyperpolarization, isotopologues, parahydrogen, SABRE hyperpolarization

## Abstract

Amino acids (AAs) and ammonia are metabolic markers essential for nitrogen metabolism and cell regulation in both plants and humans. NMR provides interesting opportunities to investigate these metabolic pathways, yet lacks sensitivity, especially in case of ^15^N. In this study, spin order embedded in p‐H_2_ is used to produce on‐demand reversible hyperpolarization in ^15^N of pristine alanine and ammonia under ambient protic conditions directly in the NMR spectrometer. This is made possible by designing a mixed‐ligand Ir‐catalyst, selectively ligating the amino group of AA by exploiting ammonia as a strongly competitive co‐ligand and preventing deactivation of Ir by bidentate ligation of AA. The stereoisomerism of the catalyst complexes is determined by hydride fingerprinting using ^1^H/D scrambling of the associated N‐functional groups on the catalyst (i.e., isotopological fingerprinting), and unravelled by 2D‐ZQ‐NMR. Monitoring the transfer of spin order from p‐H_2_ to ^15^N nuclei of ligated and free alanine and ammonia targets using SABRE‐INEPT with variable exchange delays pinpoints the monodentate elucidated catalyst complexes to be most SABRE active. Also RF‐spin locking (SABRE‐SLIC) enables transfer of hyperpolarization to ^15^N. The presented high‐field approach can be a valuable alternative to SABRE‐SHEATH techniques since the obtained catalytic insights (stereochemistry and kinetics) will remain valid at ultra‐low magnetic fields.

## Graphical abstract

Reversible hyperpolarization of ^15^N in amino acids is achieved using an iridium catalyst and parahydrogen. Ammonia competes with alanine to circumvent spontaneous deactivation of the catalyst. Hyperpolarized ^15^N NMR demonstrates reversible exchange of alanine on the SABRE catalyst, while hydride fingerprinting reveals its stereoisomerism.

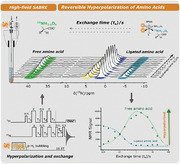

## 1 Introduction

Amino acids (AAs) and ammonia are key metabolites in the nitrogen metabolism of living organisms.^[^ ^1^, ^2^, ^3^ ^]^ Plants and microorganisms use ammonia as an intermediate to synthesize AAs.^[^ ^4^, ^5^, ^6^ ^]^ In mammals and humans, AAs exert essential roles in cell regulation,^[^ ^7^ ^]^ protein synthesis,^[^ ^8^ ^]^ metabolic processes,^[^ ^9^ ^]^ and energy production.^[^ ^10^ ^]^ Nuclear magnetic resonance (NMR) technology can serve as an excellent spectroscopic tool for the analysis of these nitrogen biochemical pathways.^[^ ^11^, ^12^, ^13^, ^14^ ^]^

Despite the versatility of NMR, the overall insensitivity associated with the inherent low spin polarization in NMR still too often prevents adequate signal detection.^[^ ^15^, ^16^ ^]^ In particular for interesting heteronuclear spins such as ^15^N and ^13^C, limited natural abundance (e.g., 0.36% for ^15^N and 1.1% for ^13^C) and a low value for the gyromagnetic ratio (*γ*) of their magnetically active isotopes creates an even higher sensitivity issue.^[^ ^2^, ^7^, ^8^ ^]^

The concept of nuclear spin hyperpolarization is a very attractive route to push NMR beyond its current boundaries.^[^ ^11^, ^17^, ^18^, ^19^, ^20^, ^21^, ^22^ ^]^ Numerous research endeavors spanning more than half a century have resulted in a wide array of physically and/or chemically orientated hyperpolarization strategies, each with its benefits and pitfalls.^[^ ^23^, ^24^, ^25^, ^26^, ^27^, ^28^ ^]^ Sensitivity enhancement is often considered the main factor for choosing a hyperpolarization strategy. But in reality, the practicality of using it under experimental conditions can even be more important for the future development of hyperpolarization applications in academia and industry.^[^ ^29^, ^30^ ^]^

In this regard, parahydrogen (p‐H_2_), the singlet spin isomer of molecular hydrogen, stands out as a cost‐effective and efficient hyperpolarization agent for enhancing spin order into interesting nuclei such as ^15^N.^[^ ^31^, ^32^, ^33^, ^34^ ^]^ P‐H_2_ can be produced cost‐effectively on demand,^[^ ^35^ ^]^ can provide a fast hyperpolarization build‐up time in seconds,^[^ ^36^ ^]^ and can be deployed under ambient experimental conditions.^[^ ^37^ ^]^ Using transition metal catalysis, p‐H_2_’s undetectable singlet magnetization is unlocked.^[^ ^38^, ^39^ ^]^ After being unlocked, the spin order can then be directed toward heteronuclear spins like ^15^N, which have slow signal relaxation, resulting in a prolonged sensitivity boost for hyperpolarized targets in solution.^[^ ^36^, ^40^, ^41^, ^42^, ^43^ ^]^

NMR studies involving AAs and/or ammonia can benefit immensely from this p‐H_2_‐enhanced sensitivity gain.^[^ ^33^, ^34^, ^44^ ^]^ Specifically in the case of AAs, various elegant methodologies using hydrogenative parahydrogen‐induced polarization (PHIP) have been proposed, yet with one common shortcoming: initial chemical modification of the AA is required, making the technology less broadly applicable.^[^ ^45^, ^46^, ^47^ ^]^ PHIP‐label and PHIP side arm hydrogenation (PHIP‐SAH) rely on unsaturated carbon‐carbon side groups functionalized onto the AA. Pairwise hydrogenation with p‐H_2_, possibly followed by cleavage of their side‐arm group, redistributes hyperpolarization across the AA.^[^ ^45^, ^48^, ^49^, ^50^ ^]^

**Figure 1:**
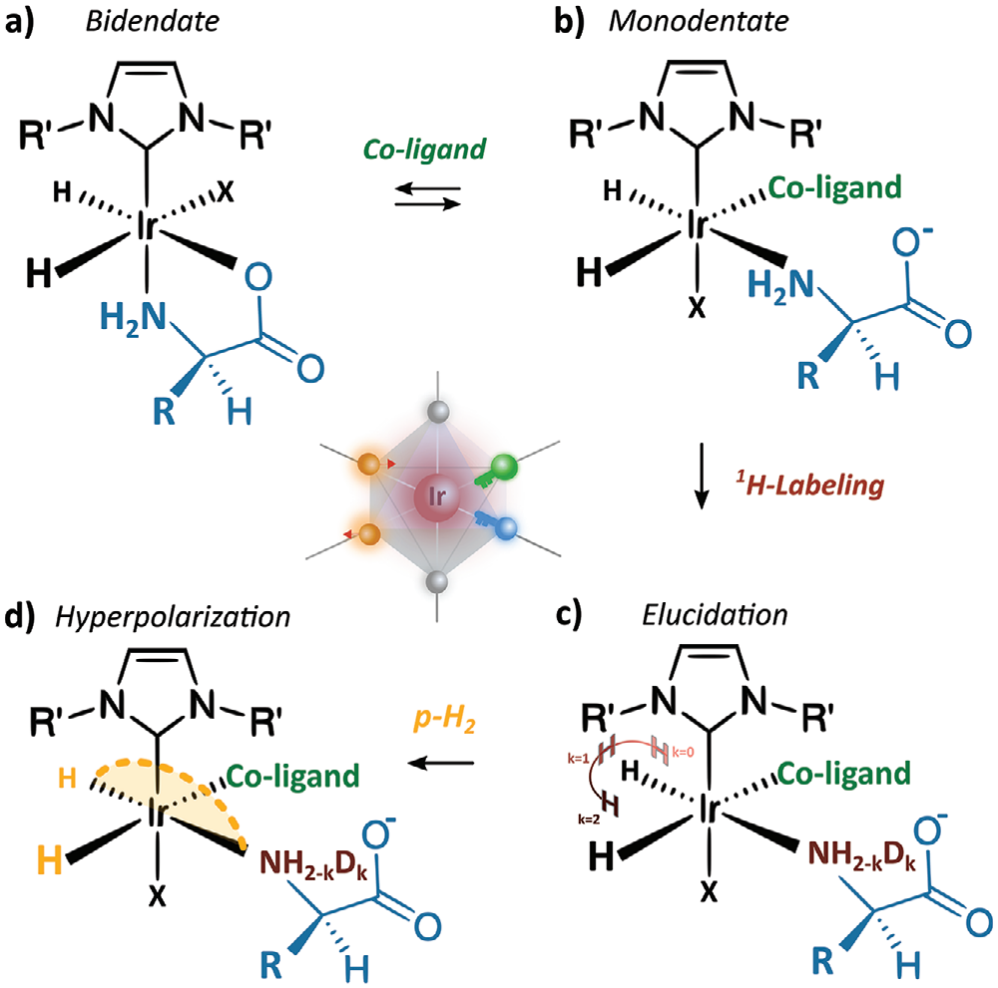
a) Thermodynamically favorable bidentate ligation of AAs to SABRE iridium catalyst inhibits their reversible hyperpolarization using p‐H_2_. b) Monodentate ligation of AAs with only the amine functionality is induced by enforcing ligand competition near the catalytic center using a co‐ligand. c) Ligand binding sites can be identified by fingerprinting the isotopic scrambling of exchangeable ^1^H and Ds on the nitrogen (N) group of the competing ligands. d) High‐field p‐H_2_ hyperpolarization of catalyst bound ^15^N nuclei is achieved using polarization transfer pulse sequences. The stabilizing ligand in the axial plane in (a)–(d) is an N‐heterocyclic carbene, while X can represent any associating ligand.

It has thus become a quest to find an alternative for hyperpolarization of unmodified AAs.^[^ ^33^ ^]^ Application of the signal amplification by reversible exchange (SABRE) strategy instantly comes to mind.^[^ ^51^, ^52^ ^]^ SABRE relies on reversible spin transfer catalysis, maintaining a balance between i) coherent spin physics involving p‐H_2_ on the active catalyst and ii) reversible chemical (ligand) exchange of the molecular target on and off the active catalyst. However, in the case of AAs, strong bidentate chelation of the metal center by the AA disrupts this balance (**Figure** **1**a) and makes hyperpolarization with SABRE exceptionally challenging.^[^ ^33^, ^34^, ^53^, ^54^ ^]^

In this report, a solution to prevent the chelation of AAs is presented to hyperpolarize ^15^N in unmodified AAs. Preventing chelation is based on the use of ammonium hydroxide buffer to progressively produce ammonia as a competing co‐ligand, encouraging monodentate ligated of alanine, a chosen representative of the AA family (Figure 1b).^[^ ^32^, ^55^, ^56^ ^]^ Experiments used p‐H_2_ and the pre‐catalyst, Ir(Cl)(COD)(IMes)^§^ together with hydrated ammonia and alanine dissolved in methanol‐d_4_ in a catalyst/alanine/ammonia ratio of 1/9.58/331, and showed that this approach minimized chelation, unlike other SABRE co‐ligandssuch as dimethylsulfoxide,^[^ ^57^ ^]^ acetonitrile,^[^ ^58^ ^]^ acetone,^[^ ^59^ ^]^ pyridine^[^ ^54^ ^]^ and additionally triphenylphosphine,^[^ ^60^ ^]^ benzylamine and taurine that where tested.

To detect and hyperpolarize AAs in association with iridium, a novel high‐field methodological approach was employed, which did not require low/high magnetic field cycling. The methodological approach consisted of two consecutive steps. First, the catalyst stereochemistry was elucidated via hydride isotopological scrambling (Figure 1c).^[^ ^61^ ^]^ Afterward, hyperpolarization of ^15^N in ligated and free (co)‐ligands was achieved via high‐field SABRE (Figure 1d).^[^ ^42^, ^43^, ^62^, ^63^ ^]^

**Figure 2:**
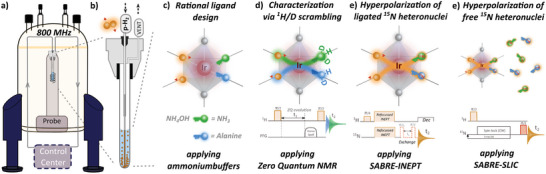
Methodological approach for allowing reversible SABRE hyperpolarization of ^15^N heteronuclei in bio‐relevant targets such as ammonia and alanine using p‐H_2_ as a source of spin‐order. a) The high‐field bubbling setup can be operated in automatic mode where p‐H_2_ activation of the SABRE Ir‐catalysts is followed by NMR pulsetrains to redistribute enhanced magnetization to heteronuclei of molecular targets inside a high magnetic field (800 MHz). No field‐cycling is therefore necessary, greatly enhancing the capabilities of continuous reaction monitoring. More information on the hardware specification can be found in Supporting Information. b) A quartz 5 mm NMR tube is modified to include a PEEK head for pressure resistant in‐ and output lines of p‐H_2_ gas. P‐H_2_ can continuously be administrated to allow for continuous SABRE‐based hyperpolarization. A combination of rational hyperpolarization catalyst design based on c) ammonia competition and d) straightforward catalyst characterization based on isotopic ^1^H/D scrambling of the ligand binding sites provides a toolset to reversible hyperpolarize less accessible targets at high magnetic field in e) ligated and f) free AAs using p‐H_2_.

This approach offered careful oversight on the present conformations of the catalysts in solution, while specific RF pulsetrains experimentally controlled the distribution of spin order to either ^15^N nuclei in alanine or ammonia. SABRE‐INEPT was used to transfer spin order from hyperpolarized hydrides to J‐coupled ^15^N heteronuclei in the bound form via hard pulses,^[^ ^64^, ^65^, ^66^ ^]^ while spin locking induced crossing (SABRE‐SLIC) was employed to hyperpolarize ^15^N heteronuclei in the free form via continuous wave irradiation on ^15^N bound substrates.^[^ ^43^, ^63^, ^67^ ^]^ The entire methodological approach for reversibly hyperpolarizing low‐y heteronuclei in challenging SABRE targets is summarized in **Figure** **2**.

## 2 Results and Discussion

### 2.1 Mechanism of Activation of IrCl(COD)IMes Using Alanine and Co‐Ligand Ammonia Substantiated by Isotopological Fingerprinting of the p‐H_2_ Derived Hydrides

Literature reports AA's deactivate Ir‐based SABRE catalysts because of chelation, bidentate ligating Ir with their carboxylic and amine functional groups (Figure 1a). The lone electron pair of carboxylic oxygens traditionally associates with the metal in the equatorial plane. Alternatively, the amine group can bind either in axial or equatorial positions, depending on physicochemical conditions such as pH, temperature, or the type of metal center.^[^ ^33^, ^34^, ^53^, ^54^ ^]^

The introduction of an aqueous NH_4_OH as an alkaline ammonium buffer into a hyperpolarization mixture containing alanine was observed to minimize catalyst deactivation and continuously hyperpolarize ^15^N in ammonia and alanine. This was seen both in catalyst‐bound and free form using the p‐H_2_ bubbling setup of Figure 2a,b. The total molar ratio (catalyst:ligand:co‐ligand) in the final solution was chosen to be 1:9.58:331 such that overconcentration of the co‐ligand forced alanine into a monodentate ligation state. A working temperature of 298 K provides a good trade‐off between reasonable exchange kinetics for inducing reversible hyperpolarization of the (co)ligands (vide infra) and suppression of bidentate configuration favored by amino acids at higher temperature (vide infra).^[^ ^54^ ^]^

First, SABRE hyperpolarization using Ir requires initial activation of the catalyst, that is, conversion of the square‐planar IrCl(COD)IMes catalyst precursor into an octahedral complex containing two hydrides, the *N*‐heterocyclic carbene and one or more substrate molecules as ligands (Figure 2c). ^1^H‐labelling of a deuterated SABRE mixture allows structure elucidation of the activated SABRE complexes by discriminating the different binding sites of ammonia and/or amine‐containing ligands based on their isotopic scrambling (Figure 2d).^[^ ^61^ ^]^

In this study, ^1^H‐labelling was performed by adding hydrated ammonia (NH_4_OH) to a mixture containing: i) IrCl(COD)IMes, ii) p‐H_2_, iii) alanine, and iv) deuterated methanol‐d_4_. The resulting distribution of hydrides trans to the functional binding sites reveals the isotopic scrambling of the substrates and thus the structure of different mixed‐ligand complexes formed upon activation of the IrCl(COD)IMes precursor.

P‐H_2_ activation was performed in situ in a 5 mm quartz NMR tube using the high‐field p‐H_2_ bubbling setup described in Figure S1, Supporting Information, and highlighted in Figure 2. The high‐field p‐H_2_ bubbling setup circumvents the need for interruptive field cycling for catalytic structure determination.^[^ ^68^ ^]^

**Figure 3:**
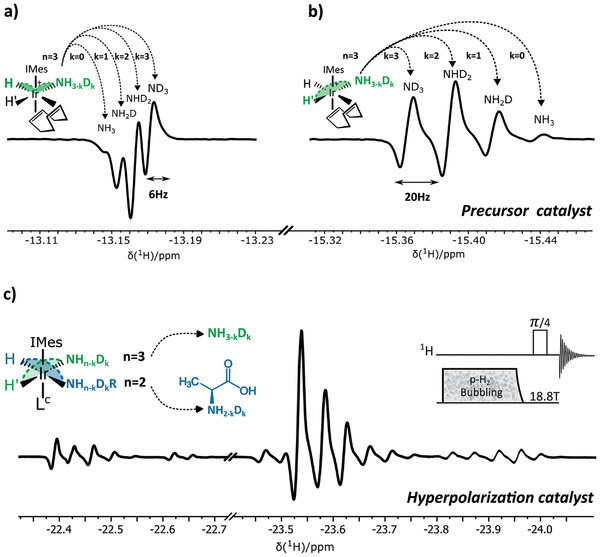
Hydrides originating from oxidative addition of p‐H_2_ to the catalytic center where alanine is competing with ammonia for ligation to iridium are visible after a *π*/4 pulse. a) *Cis*‐positioned hydrides in respect to ammonia in [Ir(IMes)(H_2_)(COD)(S_eq_)]Cl^−^ only experience a slight chemical shift variation of each ammonia isotopologues (6 Hz) and are visible at *δ* −13.15 ppm. Trans‐positioned hydrides in respect to ammonia in [Ir(IMes)(H_2_)(COD)(S_eq_)]Cl^−^ experience a much larger chemical shift variation of each ammonia isotopologues (20 Hz) and are visible around *δ* −15.4 ppm. c) The hydride region at *δ* −22.35 to −24 ppm corresponds to an active [Ir(IMes)(H_2_)(S_eq1_S_eq2_S_ax_)]Cl^−^ SABRE catalyst where competition of alanine and ammonia gives rise to multiple catalyst configuration. 2D zero‐quantum ^1^H‐NMR applied to these hydrides (Figure 4) reveals the competing ligand behavior and also the resulting octahedral catalyst conformations.

Following the initial activation with p‐H_2_, two isotopologically scrambled antiphase hydride resonances emerge centered around *δ* 13.15 ppm and *δ* 15.4 ppm in the 1D spectrum recorded using a *π*/4 excitation pulse (**Figure** **3**). This spectroscopic region (Figure 3a,b) reveals the hydride signals of an intermediary complex with the general formula [Ir(IMes)(H_2_)(COD)(S_eq_)]Cl^−^ (Figure S2,II, Supporting Information); with S_eq_ representing a substrate bound in the equatorial plane. The isotopically scrambled resonances consisting of four isotopologues separated by 20 Hz and centered around *δ* −15.4 ppm (Figure 3b) readily reveal the configuration of the intermediate complex. These resonances correspond to ammonia positioned trans to this hydride. Ammonia exhibits a maximum of four isotopic variations (—ND_3_, —NHD_2_, —NH_2_D, —NH_3_). In contrast, the primary amine of alanine as an equatorial ligand would give rise to a maximum of three isotopologues (—ND_2_, —NHD, —ND_2_).

At equilibrium, the relative concentrations of the different isotopologues are described by a binomial distribution (Equation (1)):

(1) $$x=\left(\begin{array}{c} n\\ k \end{array}\right)d^{k}{\left(1-d\right)}^{n-k}$$

with *x* being the molar fraction of each isotopologue, *n* the total number of deuterons/protons possible for the functional group, *k* the number of deuterons specific to each isotopologue, and *d* the percentage of deuterons in total.^[^ ^69^, ^70^ ^]^ Figure S4, Supporting Information, shows a plot of the fractional isotopologue distribution as a function of the degree of protonation of ammonia and alanine respectively.

The four hydride resonances centered around *δ* −13.15 ppm (Figure 3a) again are associated with ammonia. Their moderate isotopic shifting of 6 Hz reveals these resonances represent the hydride positioned cis to ammonia. The spectrum shown in Figure 3a,b thus reveals the freshly activated solution contains mainly one Ir complex, [Ir(IMes)(H_2_)(COD)(NH_3‐k_D_k_)]Cl^−^. Ammonia in the form of NH_3‐_ *_k_* D*_k_* is thus responsible for the initial activation of the precursor complex (additional proof using ^15^N‐labelled ammonia is provided in Figure S5, Supporting Information), leaving alanine out of the catalytic reaction pathway. This is already a first step to avoid deactivation by the AA during the preparation of the SABRE active complex.

Finally, the antiphase character of the hydride resonances finds its origin in the chemical inequivalence of the hydride couple in [Ir(IMes)(H_2_)(COD)(NH_3‐_ *_k_* D*_k_*)]Cl^−^, resulting from the ligation of co‐ligand ammonia. The magnetization observed for the hydride resonances (denoted with ${\hat{I}}_{1}\;$and with ${\hat{I}}_{2}$) is generated by spontaneous singlet |*S*_0_〉 → |*T*_0_〉 triplet mixing of the asymmetric hydride couple at high‐field.^[^ ^32^, ^71^, ^72^ ^]^ This unlocks the singlet polarization and converts it into triplet longitudinal two‐spin order ${\hat{I}}_{1z}{\hat{I}}_{2z}$ (Figure S3a, Supporting Information). Following a *π*/4 read‐out pulse (pulsetrain depicted in Figure 3) this gives rise to the observation of antiphase ${\hat{I}}_{1x}{\hat{I}}_{2z}\;+{\hat{I}}_{1z}{\hat{I}}_{2x}$ magnetization. Figure S2, Supporting Information, demonstrates the complete cycle generating hyperpolarized magnetization from parahydrogen starting with the activation of the catalytic complex followed by a sequence involving the spin transfer catalysis, hydrogen, as well as substrate exchange.

### 2.2 Hydride Zero Quantum Coherences Identify Octahedral SABRE Catalyst Complexes

Following initial activation of the catalyst precursor, the intermediate complex ([Ir(IMes)(H_2_)(COD)(NH_3‐_ *_k_* D*_k_*)]Cl^−^) is transformed into an activated octahedral complex with the general formula [Ir(IMes)(H_2_)(S_eq,1_)(S_eq,2_)(S_ax_)]Cl^−^ (Figure S2,III, Supporting Information). This conversion can be monitored in the hydride region between ‐25 and *δ* ‐22 ppm (Figure 3c). While the isotopological scrambled hydride resonances of the [Ir(IMes)(H_2_)(COD)(NH_3‐_ *_k_* D*_k_*)]Cl^−^ intermediate were easily assigned from their 1D ^1^H spectra, the array of resonances observed between *δ* −25 and −22 ppm is way too complex for analysis using 1D spectra only. Visualizing the isotopic scrambling using 2D zero‐quantum NMR spectroscopy (2D‐ZQ‐NMR), structure elucidation of the complexes present in the hyperpolarization mixture however becomes straightforward.^[^ ^54^, ^61^ ^]^

**Figure 4:**
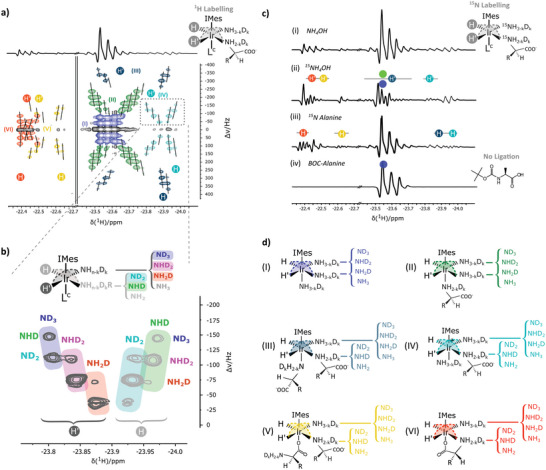
Analysis of the association behavior of hyperpolarization targets alanine and in competition with ammonia to the iridium SABRE catalysts. a) ^1^H‐labelling of the (co)ligand binding sites becomes projected onto the corresponding hydrides and can be visualized via hyperpolarized 2D zero‐quantum ^1^H‐NMR. The vertical axis of the 2D ZQ plot projects the ZQ evolution rate of all hydride couples, highlighting the binomial distribution of isotopologues of the ligand binding sites and reveals the SABRE catalyst configuration. Two distinct hydrides regions around *δ* −22.5 ppm and −23.75 ppm are attributed to differing axial ligation behavior of either an oxygen or nitrogen functionality respectively. Notice that even though they are not always equally well resolved, the top and bottom parts of these patterns (Δ*v* around −300 to 0 Hz and 300 to 0 Hz, respectively) represent the same hydride. b) A zoomed‐in version of the ZQ fingerprint of motif IV is presented. c) ^15^N‐labelling of the (co)ligand binding sites becomes projected onto the corresponding hydrides and is visualized via hyperpolarized ^1^H‐NMR. i) Traditional hydride response of alanine in competition with ammonia after p‐H_2_ activation of the Ir‐catalyst at high‐field. ii) Hydride response after ^15^N‐labelling of ammonia. The affected hydrides are highlighted by a grey line and correspond to the elucidation provided by the 2D ZQ plot. iii) Hydride response after ^15^N‐labelling of alanine. The effected hydride regions are highlighted by a grey line and agree with the elucidation based on the 2D ZQ plot. iv) Hydride response after BOC‐capping of the primary amine of alanine to block N‐based ligation to the Ir‐center. d) The iridium catalyst conformations (I–VI) are derived from the results obtained in (a), (b), and (c) and rationalize the competing behavior of the ammonia and alanine hyperpolarization targets.

In the octahedral SABRE complex, ZQ coherences created from the longitudinal triplet magnetization of a hydride couple evolve under their chemical shift difference.^[^ ^61^ ^]^ Such chemical shift difference is generated when different ligands or different isotopologues of the same ligand occupy the equatorial positions (trans to the hydrides). Consequently, the rate of ZQ evolution becomes dependent on the chemical and isotopological composition of the complex (**Figure** **4**b). Plotting the ZQ evolution rate of the hydrides versus their chemical shift in a 2D ZQ plot readily separates out the hydride couples for each unique structure. This approach also provides an isotopological fingerprint assisting to discriminate all substrates (ammonia and/or alanine) bound in each complex.^[^ ^54^, ^61^ ^]^ A detailed description of the pulse sequence and experimental conditions allowing the construction of such 2D ZQ plots is provided in the experimental procedure details available in the Supporting Information (Figures S3, S6, S12, Supporting Information).

### 2.3 Ammonia and Alanine Compete for the Same Ligand Positions on the Ir Hyperpolarization Catalyst

Figure 4a shows the total 2D ZQ plot corresponding to the 1D hydride spectrum shown in Figure 3c. Hydride signals with identical ZQ evolution rates belong to a single hydride couple and thus to a unique catalyst configuration. The plot readily highlights the presence of 6 different complexes (I–VI). The complexes observed using the 2D‐ZQ plot are all confirmed by the independent ^15^N‐labelling study presented in Figure 4c. Detailed plots of their hydride spectra are shown in Figure S7, Supporting Information. Both methods, isotopological scrambling and ^15^N labelling provide the same information. This highlights the reliability and strength of the significantly faster and less expensive isotopological scrambling method. In addition, this method is always available, also in situations where ^15^N‐labelling is not an option. In the systems where ^15^N ammonia was used (Figure 4c,ii), the additional J‐coupling between the hydride and ^15^N reveals the resonances corresponding to a hydride positioned trans to ammonia in the equatorial plane. In the systems containing ^15^N‐labelled alanine (Figure 4c,iii), the J‐coupling between the hydride and ^15^N readily identifies resonances corresponding to a hydride positioned trans to alanine in the equatorial plane.

#### 2.3.1 Equatorial Ligands

Combined with ^15^N‐labelling results, the presence of multiple isotopologues for every hydride resonance in the chemical shift region between *δ* −22.35 and −24 ppm univocally reveals all complexes in the mixture (Figure 4,I–VI) contain N‐bonded ligands, ammonia, and/or N‐bonded alanine, in their equatorial plane. Complexes containing an oxygen‐bonded ligand trans to the hydrides would be shifted to a chemical shift range around *δ* −28 ppm (Figure S8, Supporting Information) and were previously demonstrated to be inactive in SABRE hyperpolarization.^[^ ^54^ ^]^ The near absence of such signals at room temperature readily indicates the addition of ammonia suppresses the formation of unwanted bidentate AA complexes binding to Ir as depicted in Figure 1a.

#### 2.3.2 Axial Ligands

In contrast to equatorial ligands, axial ligands only exert minimal influence on the hydrides situated in the equatorial plane. Isotopological scrambling in the axial position did not seem to affect the equatorially positioned hydrides. The association of different functional groups in the axial position has however been shown to induce a chemical shift translation of equatorial motifs. This effect has been ascribed to both steric and electronic effects.^[^ ^61^, ^73^ ^]^ Figure 4a reveals two separated hydride regions, hinting at the presence of different ligands in the axial positions of the complexes associated with motifs I–IV (from *δ* −23.45 to −24.00 ppm) and motifs V–VI (from *δ* −22.35 to −22.65 ppm). The chemical shift range from *δ* −23.45 to −24.00 ppm shows the hydride couple fingerprints of complexes with nitrogen functional groups in their axial positions (vide infra).^[^ ^32^, ^61^ ^]^ ln the present system, the hydride resonances occurring in this region are associated with complexes containing either ammonia or N‐bonded alanine in their axial position. The chemical shift range from *δ* −22.35 to −22.65 ppm in Figure 4a shows fingerprints of complexes with oxygen functional groups in their axial positions (vide infra).

### 2.4 Identifying SABRE Complexes Containing Ammonia and Alanine

#### 2.4.1 Motifs I–II

Replacing alanine with N‐(tert‐butoxycarbonyl)‐alanine instantly reveals which resonances belong to complexes containing N‐bonded alanine. N‐(tert‐butoxycarbonyl) (BOC) capping deactivates the amine functionality of alanine preventing ligation. Comparing the hydride spectra of systems with alanine (Figure 4c,i) with those for a system with BOC‐capped alanine (Figure 4c,iv) readily demonstrates that only motif I represents a complex without N‐bonded alanine. In the system containing BOC‐capped alanine indeed only motif I remains (Figure 4d,I).

Vaneeckhaute et al. previously assigned the ZQ motifs of complexes containing two ammonia ligands in the equatorial plane,^[^ ^61^ ^]^ thus allowing the assignment of fingerprints I and II appearing around *δ* −23.5 ppm in Figure 4a. Fingerprint I (marked in blue) shows the fingerprint typical for a complex with two equatorially bound ammonia ligands and a third ammonia in the axial position (Figure 4d,I).^[^ ^61^ ^]^ Fingerprint II, shown in green, also reveals four isotopologues on each side and can be assigned to a complex with two equatorially bound ammonia ligands and an N‐bonded alanine in the axial position (Figure 4d,II). Fingerprint II disappeared when alanine was replaced by BOC‐capped alanine, thus confirming the identification of the axial ligand while the identity of the equatorial ligands (ammonia) is revealed by the 2D ZQ plot. ^15^N‐labelled hydrated ammonia confirmed the identification of the equatorial ligands in the complexes represented by identity fingerprints I and II. By using ^15^N‐labelled ammonia also the J‐coupling between the hydrides and the ^15^N nuclei is revealed (Figure 4c,ii) for the hydride couples in both motifs I and II. The larger chemical shift difference between signals from H and H’ in motif II as compared to motif I is attributed to the steric influence of N‐bonded alanine as compared to ammonia in the axial position.

#### 2.4.2 Motifs III–IV

The catalyst configurations corresponding to motifs III to IV were analyzed in detail based on the ZQ‐plot shown in Figure 4a, again combined with 1D spectra acquired on catalysts mixtures containing either ^15^N‐labelled ammonia or ^15^N‐labelled alanine (Figure 4c). Motif III (Figure 4a, dark blue) and motif IV (Figure 4a, light blue) represent two complexes containing both ammonia and N‐bonded alanine in their equatorial plane. As highlighted in the zoomed‐in version of motif IV in Figure 4b, the left side of motifs III and IV, marked with H′, shows a 3 × 2 pattern. At the ^1^H/D ratio in the system, this isotopological distribution in combination with its chemical shift reveals a hydride positioned trans to ammonia. The corresponding right‐shifted hydride resonances belonging to complexes III and IV, marked with H in Figure 4a, are split twofold, yielding a 2 × 3 motif. Following the method discussed in Vaneeckhaute et al.,^[^ ^61^ ^]^ this pattern can be assigned to a hydride trans to a primary amine. Note that the ^1^H content of the system only allows for partial isotopological scrambling. Fully isotopological scrambled fingerprints for a hydride trans to ammonia and a primary amine should respectively result in a 4×3 and 3×4 motif. However, for the quantity of protons used in our chemical system (see Figure S4, Supporting Information), the concentration of the fully protonated isotopologues falls below the detection limit, reducing the ammonia and amine fingerprints to a 3 × 2 and 2 × 3 pattern, respectively. Figure S4, Supporting Information, provides more details on the dependence of the isotopological patterns on the ^1^H/D ratio in the system. The identity of the equatorial ligands in the complexes is represented by fingerprint III and is again verified by replacing hydrated ammonia and alanine with their ^15^N‐labelled versions (in Figure 4c,ii,iii, respectively). Analogous to what was observed for motifs I and II, the larger chemical shift difference between signals from H and H’ witnessed in motif IV as compared to motif III is attributed to the steric influence of N‐bonded alanine in the axial position. The suggested octahedral configurations for motifs III and IV in Figure 4a are presented in Figure 4d,III,IV. These will be crucial for the hyperpolarization of free alanine.

#### 2.4.3 Motifs V–IV

Motif V (yellow) is highly similar to motif IV (light blue) but shifted from the chemical shift region with an N‐bonded axial ligand to the region associated with O‐bonded axial ligands. Motif V is thus assigned to a complex with ammonia and N‐bonded alanine occupying the equatorial ligand positions and O‐bonded alanine occupying the axial position (Figure 4d,V). In motif VI (red), the patterns revealing ammonia and N‐bonded alanine are mirrored as compared to motif V. As can be seen in Figure 4a, the left side of the motif (H) represents a hydride positioned trans to alanine, while the right side (H′) represents a hydride positioned trans to ammonia. The axial ligand is suggested to be a carboxyl functionality of bidentately ligated alanine also occupying a position in the equatorial plane. This could induce the observed mirroring in the hydride couples. Such bidendate ligation of course renders this configuration not susceptible to SABRE hyperpolarization.

The suggested octahedral configurations for every motif are shown in Figure 4d. Identification of the axial position remains difficult, but the results indisputably demonstrate monodentate ligation of alanine at 298 K in the presence of ammonia as a co‐ligand. This is only the first, yet crucial step toward SABRE‐induced hyperpolarization of AAs.

### 2.5 High‐Field SABRE Hyperpolarization of Ligated ^15^N‐Heteronuclei

Following elucidation of the structure of complexes I–VI (Figure 4), these complexes can now be used to attempt SABRE hyperpolarization of ^15^N in ammonia and alanine, in our case transferring spin polarization from the hyperpolarized hydrides to ^15^N of ligated ammonia or alanine at high‐field conditions.^[^ ^74^ ^]^ Hyperpolarization of non‐ligated ammonia and alanine requires initial transfer of enhanced ^1^H spin polarization of the hydrides to ligated ^15^N, followed by the chemical exchange of the molecules within the lifetime of the hyperpolarization at a rate suitable to build up ^15^N hyperpolarization.^[^ ^43^ ^]^

At 18.8 T, there are no level anticrossings (LACs) that enable spontaneous coherent SABRE polarization transfer from p‐H_2_ to a target.^[^ ^27^, ^75^ ^]^ Transfer of polarization from the hyperpolarized hydrides on the complex to ^15^N in the target must be forced using RF‐driven SABRE techniques (Figure 2e,f).^[^ ^68^ ^]^

**Figure 5:**
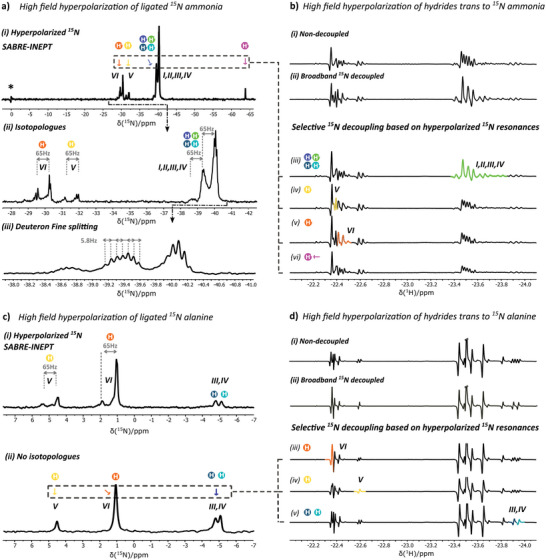
Hyperpolarization of ^15^N nuclei of ligated ammonia and alanine targets in high‐field conditions fueled by the spin‐order embedded in p‐H_2_. Hyperpolarization is transferred from p‐H_2_ to the substrates via a J‐based INEPT polarization transfer scheme (SABRE‐INEPT) that can be found in Figure S12, Supporting Information. Characterization of the ^15^N resonances was derived from selective ^15^N decoupling during hydride acquisition. This correlation method directly couples ^15^N resonances with hyperpolarized hydride signals to which an assignment already is available. a) Hyperpolarized ^15^N resonances arising from ligated ^15^N‐labelled ammonia in competition with unlabeled alanine. i) Two distinct peak regions around *δ* −30 ppm and −40 ppm are observed in congruence with the hydride signal pattern (*δ* −22.5 ppm vs −23.75 ppm). The peak at *δ* 0 ppm is from free ammonia. ii) Isotopologues of ammonia are also observed in ^15^N with a 65 Hz separation, while in iii) an additional deuteron (*I* = 3/2) fine spitting pattern seems present. b) ^15^N resonances of ligated ammonia are related to i) the hydride resonances ii) based on broadband decoupling, iii–vi) combined with selective ^15^N decoupling. c) Hyperpolarized ^15^N signals are arising from ligated ^15^N‐labeled alanine in competition with unlabeled ammonia i) in presence of ^1^H/D scrambling and ii) without the effect of ^1^H/D scrambling. d) ^15^N resonances of ligated alanine are related to i) the depicted hydride resonances ii) based on broadband decoupling, iii–v) combined with selective ^15^N decoupling. The assigned ^15^N resonances can be used as starting point for the spin‐locking induced high‐field hyperpolarization experiments. Temporary RF spin‐locking of these ^15^N resonances allows coherent spin order transfer from p‐H_2_ to free ammonia and alanine substrates.

Using the SABRE‐INEPT sequence in a system containing the iridium catalyst activated in the presence of ^15^N‐labelled ammonia or ^15^N‐labelled alanine, spin order from the hyperpolarized hydrides on the SABRE complex was easily transferred to ^15^N heteronuclei in ligated ammonia and alanine (**Figure** **5**a,b).^[^ ^64^ ^]^ To facilitate the assignment of the hyperpolarized ^15^N signals, SABRE‐INEPT was performed in systems containing either ^15^N‐labelled ammonia and natural abundance alanine (i, Figure 5a) or ^15^N‐labelled alanine and natural abundance ammonia (ii, Figure 5c). For a detailed description of the experimental parameters used to generate the hyperpolarized ^15^N spectra presented in Figure 5, the reader is referred to the experimental section of the Supporting Information.

#### 2.5.1 (i) ^15^N‐labelled Ammonia and Alanine at ^15^N Natural Abundance

Performing SABRE‐INEPT in systems containing ^15^N‐labelled ammonia in combination with alanine at ^15^N natural abundance gives rise to 3 sets of hyperpolarized ^15^N resonances, centered around *δ* −30, −40, and ‐64 ppm (Figure 5a,i). Applying selective ^15^N decoupling, the ^15^N‐induced fine splitting of the hydride signals is selectively removed from the hydride trans to the decoupled nitrogen group. This enables a direct correlation between hyperpolarized ^15^N and hyperpolarized hydride signals (Figure 5b). Using the pulse sequence presented in Figure S12a, Supporting Information, each of the hyperpolarized ^15^N resonances shown in Figure 5a was correlated to its corresponding set of hyperpolarized hydride signals in Figure 5b. When the ^15^N region around *δ* −40 ppm was selectively decoupled, the hydride region around *δ* −23.5 ppm was affected. This region contains the resonances of hydrides positioned trans to ammonia in complexes containing an N‐bonded ligand in the axial position (Figure 4d,I–IV). Selective decoupling in the ^15^N region around *δ* −30 ppm affected the hydride resonances around *δ* −22.5 ppm, corresponding to hydrides positioned trans to ammonia in complexes with an O‐bonded ligand in the axial position (Figure 4d,V,VI). The chemical shift translation between the two ^15^N regions is caused by the presence of different axial ligands in the octahedral complex. When either ammonia or alanine are binding with their N‐functionalities the ^15^N resonance of equatorial bounded ammonia appears at *δ* −40 ppm. In case the O‐functionality of alanine is binding (both in bidentate and monodentate form) the ^15^N resonance of equatorial bounded ammonia appears at *δ* −30 ppm. Selective irradiation on the final and most upfield ^15^N resonance at *δ* −64 ppm, did not influence the hydride region corresponding to the active SABRE catalyst (Figure 5b,vi). Instead, the precursor catalyst region visible between *δ* ‐13.15 ppm and *δ* ‐15.4 ppm was affected (Figure S9, Supporting Information). The ^15^N resonance at *δ* −64 ppm thus corresponds to the catalyst structure depicted in Figure 3a.

Similar to the isotopological distributions observed in the ^1^H spectra of the SABRE complexes, isotopological distributions can also be observed in the hyperpolarized ^15^N spectra. Figure 5a,ii reveals a splitting of the catalyst‐bound ^15^N ammonia resonances by 65 Hz induced by the isotopological distribution. The additional fine splitting (5.8 Hz) (Figure 5a,iii) is attributed to a *J*‐coupling with the deuterons attached. Literature reports the *J*_15N‐D_ coupling in ammonia to be 11.3 Hz.^[^ ^70^ ^]^ The discrepancy with the value here observed is still under investigation. To boost signal to noise even more in ^15^N, deuteron decoupling was applied during all later experiments to suppress this fine splitting pattern.

#### 2.5.2 (i) ^15^N‐labelled Alanine and Ammonia at ^15^N Natural Abundance

When ^15^N‐labelled alanine is used in combination with ammonia at ^15^N‐natural abundance, SABRE‐INEPT (detailed parameters found in the Supporting Information) reveals hyperpolarized ^15^N resonances in two regions (Figure 5c,i): *δ* 0–7 ppm and around *δ* −5 ppm. These regions are assigned to motifs V and VI (oxygen in the axial position) and motifs III and IV (nitrogen in the axial position), respectively.

Direct correlation between ^15^N resonances of bound alanine and hyperpolarized hydride signals was performed analogously as for ammonia, applying selective ^15^N decoupling during hydride observation. Suppressing ^1^H/D isotopologue formation by minimizing ^1^H‐labelling assisted to simplify the ^15^N decoupled hydride resonances. Minimizing ^1^H‐labelling was achieved using a deuterated ammonium buffer (ND_4_OD) source. The hyperpolarized ^15^N resonances of ligated alanine without ^1^H/D scrambling are shown in Figure 5c,ii.

When the ^15^N region between *δ* 0 and 7 ppm was selectively decoupled, the hydride region between *δ* −22.35 and −22.65 ppm was affected. This region corresponds to hydrides positioned trans to alanine in complexes with an O‐bonded axial ligand. More specifically, selective decoupling in the ^15^N region at *δ* 1 ppm affected the hydride resonances around *δ* −22.35 ppm associated with complex VI in Figure 4d. In turn, selective ^15^N decoupling at *δ* 5 ppm highlights hydrides at *δ* −22.6 ppm, associated with complex V in Figure 4d. Irradiating at *δ* −5 ppm on the ^15^N channel during the acquisition of the hydride signals, the hydride region at *δ* −23.9 ppm, corresponding to complex III and IV (Figure 4d), was affected.

### 2.6 High‐Field SABRE Hyperpolarization of Free ^15^N‐Heteronuclei

#### 2.6.1 SABRE‐INEPT with Exchange Delay

Having demonstrated ^15^N hyperpolarization of ligated ammonia and alanine substrates, hyperpolarization of the free substrates can be attempted. The experimental freedom to redistribute hyperpolarization to both ammonia and AA bio‐markers inside the spectrometer can itself advance biochemical reaction monitoring, even enabling hyperpolarized multidimensional NMR analysis due to the long inherent T_1_ relaxation time of ^15^N.^[^ ^43^, ^44^, ^63^, ^64^, ^76^ ^]^

**Figure 6:**
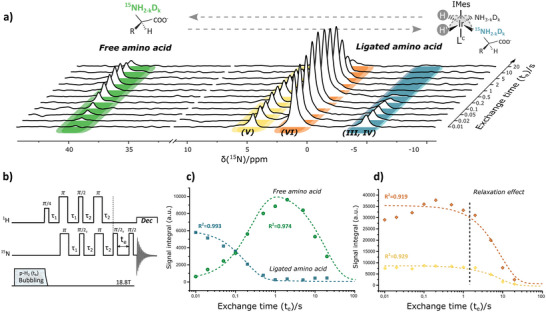
Evolution of ^15^N hyperpolarization induced at high‐field (18.8 T) of ligated and free AAs monitored via increasing the exchange time (*t*_e_) of an adapted SABRE‐INEPT pulsetrain. a) Overview of the ligated and free hyperpolarized ^15^N resonances of alanine in function of increasing exchange time (*t*_e_). For each exchange time, p‐H_2_ was bubbled for a duration of 5 s into a hyperpolarization mixture with ^15^N‐labelled alanine, deuterated ammonium buffer and Ir‐catalyst at high field. b) Adapted SABRE‐INEPT pulsetrain: i) after a refocused INEPT block, hyperpolarization is protected from fast transversal relaxation on the catalyst via a first *π*/2 storing magnetization longitudinally, ii) during the exchange time (*t*_e_) hyperpolarized targets can dissociate for building‐up of magnetization in free substrates, and iii) final hyperpolarization is monitored by a second *π*/2 read‐out pulse. c) Plot of the evolution of the hyperpolarization intensity of ^15^N in respect to the exchange time (logarithmic scale) correlating the magnetization stored in ligated alanine in complex III and IV (*δ* −5 ppm) to the main hyperpolarization stored in the free form (around *δ* 40 ppm). Data is fitted by a least square non‐linear regression using Equations (2) and (3) that describe the evolution of magnetization corresponding to ligated and free hyperpolarized alanine. d) Plot of the evolution of the hyperpolarization intensity of ^15^N in respect to the exchange time (logarithmic scale) for ligated alanine in complexes V (*δ* 5 ppm) and VI (*δ* 1 ppm).

This requires initial hyperpolarization of the ligated substrate in combination with a sufficiently fast chemical exchange of substrate to enable observation of the hyperpolarized ^15^N nuclei in the free substrates (**Figure** **6**a). To enable observation of free hyperpolarized substrates using SABRE‐INEPT, a delay (*t*_e_) must be implemented between the initial transfer of polarization to the ligated substrate and the 90° readout pulse (Figure 6b). Hyperpolarized ^15^N in the free substrate could thus be observable provided the chemical exchange of the substrate is fast enough compared to relaxation in its ligated state.

In Figure 6, the exchange behavior for alanine in different complexes (III–VI) is observed by adjusting the delay time (*t*_e_) and measuring the corresponding hyperpolarized ^15^N resonances. Increasing the exchange time (in this case from 10 ms to 20 s), hyperpolarization is expected to become gradually stored in the free form of the AA (at *δ* 40 ppm).

The evolution of the hyperpolarization intensity in both ligated as well as in the free form of the ^15^N resonances are plotted in Figure 6c,d. Hyperpolarization in the free AA, although still moderate in enhancement (an order of magnitude), increases with exchange time. On the other hand, a concurrent decrease of hyperpolarization is observed mostly in ligated alanine of complex III and IV (Figure 6c) proving exchange due to monodentate ligation once more. The evolution of magnetization in this SABRE‐adapted form of high‐field exchange spectroscopy is governed by a two‐sites exchange process^[^ ^77^ ^]^ in which the magnetization of the ligated and free hyperpolarized substrates can be described by Equations (2) and (3), respectively:

(2) $$I^{L}\left(t_{e}\right)=\eta_{2}\left(e^{-\lambda_{1}t_{e}}+\eta_{1}e^{-\lambda_{2}t_{e}}\right)I^{L}\left(0\right)$$

(3) $$I^{F}\left(t_{e}\right)=\eta_{2}\left(-e^{-\lambda_{1}t_{e}}+e^{-\lambda_{2}t_{e}}\right)I^{L}\left(0\right)$$

where *I* ^L^ is the magnetization intensity of the ligated target, *I* ^F^ is the magnetization intensity of the free target, *λ*_1_ is a characteristic rate constant incorporating both the relaxation and the dissociation rate of the ligated target, *λ*_2_ is a characteristic rate constant incorporating both the relaxation and the association rate of the free target and *η*_1_ and *η*_2_ are weight factors for the influence of each exponential decay. In the ideal case, the contributions of both exchange and relaxation can be discriminated,^[^ ^77^ ^]^ yet we were only allowed to fit the values for *λ*_1_ and *λ*_2_. These provide an indication of the combined exchange and relaxation characteristics between the different catalyst configurations (**Table** **1**).

**Table 1:** Fitting values with standard deviation errors for the exchange data in Figure 6 fitted by the corresponding Equations (2) and (3) describing the exchange of hyperpolarized targets in SABRE conditions

Magnetization	*λ*_1_ [s^−1^]	*λ*_2_ [s^−1^]	*η*_1_ [s^−1^]	*η*_2_ [s^−1^]
Ligated III, IV	4.87 ± 0.38	/	0	0.99 ± 0.03
Ligated V	0.12 ± 0.022	/	0	1.19 ± 0.05
Ligated VI	0.12 ± 0.020	/	0	1.14 ± 0.04
Free	4.04 ± 0.54	0.06 ± 0.008	/	1.76 ± 0.08

Instantly, the large *λ*_1_ characteristic rate constant for complexes III and IV is noticeable. The similar magnitude of *λ*_1_ observed for the evolution of magnetization in the free form intercorrelates complex III and IV as the active complexes for reversible hyperpolarization. In contrast, *λ*_1_ for complex V and VI is 50 times slower. Since the value of *λ*_1_ both projects dissociation and relaxation effects in ligated substrates, dissociation of alanine in complex V and VI is certainly much slower. Their contribution to hyperpolarizing free AAs is therefore negligible.

These results are expected since complexes V and VI were already appointed less susceptible to SABRE hyperpolarization due to the possibility of bidentate ligation based on their configuration. Also note the absence of *λ*_2_ in fitting the ligated magnetization in each complex. The reason is twofold here. For complex III and IV, decay of magnetization is so fast, slower rate processes will not be visible due to limited leftover magnetization. For complex V and VI, the influence of *λ*_2_ is absent since without dissociation occurring, this characteristic rate constant unique to free substrates cannot influence the magnetization decay. Each of the resulting fits and their *r*‐squared values is displayed on top of Figure 6,c,d.

Also, the Pearson correlation (*r*_p_) between the evolution of the hyperpolarization intensity of ligated alanine (decreases with increasing exchange time) and that of free alanine (increases with increasing exchange time) enables us to trace back the main complexes responsible for hyperpolarization. In this case, hyperpolarization in free form is fueled mainly by complex III and IV with an *r*_p_ of −0.992, while for complex V and VI, *r*_p_ is only −0.42 and −0.46, respectively. After an exchange time of 3 s, relaxation effects tend to decrease magnetization. The same phenomenon is apparent for hyperpolarization in the free form.

#### 2.6.2 Spin‐Lock Induced Crossing (SLIC)‐SABRE

**Figure 7:**
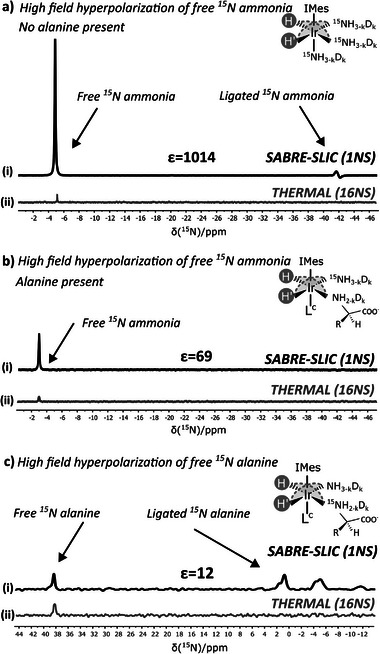
Hyperpolarization of ^15^N nuclei of free ammonia and alanine substrates in high‐field conditions using p‐H_2_ and the spin‐locking pulsetrain (SABRE‐SLIC) highlighted in Figure S12, Supporting Information. a) Hyperpolarized ^15^N resonances i) arising from free ^15^N‐labelled ammonia without presence of competing alanine targets. The equilibrium signal for free ^15^N‐labelled ammonia after 16 scans is given in (ii). b) Hyperpolarized ^15^N signals arising from free ^15^N‐labelled ammonia (I) in presence of competing alanine targets. The slight chemical shift from *δ* −5 ppm to *δ* −3 ppm of free ammonia can be attributed to a pH difference after adding alanine. The equilibrium signal for free ^15^N‐labelled ammonia after 16 scans is given in II. c) Hyperpolarized ^15^N signals arising from free ^15^N‐labelled alanine (I) in competition with ammonia. The equilibrium signal for free ^15^N‐labelled alanine after 16 scans is given in II.

For hyperpolarization of free substrates, spin‐locking‐induced anticrossings can provide an improved option to transfer spin polarization.^[^ ^63^ ^]^ When utilizing radiofrequency irradiation to perform spin‐locking of ligated substrates, the presence of level anticrossings can be leveraged to facilitate spin polarization transfer at high field throughout the entire spin‐lock duration. This mechanism is related to polarization transfer in low‐field SABRE applications, where the polarization transfer occurs without spin‐lock. In contrast to SABRE‐INEPT, SABRE‐SLIC can only be achieved if the ^15^N chemical shifts of the bound substrates are known.^[^ ^78^ ^]^ Similar to the approach used for hyperpolarizing ^15^N nuclei in ligated substrates using SABRE‐INEPT, SABRE‐SLIC transfer was performed in systems containing either ^15^N‐labelled ammonia (**Figure** **7**a), ^15^N‐labelled ammonia and natural abundance alanine (Figure 7b) or ^15^N‐labelled alanine and natural abundance ammonia (Figure 7c). The spin‐locking pulsetrain employed here (Figure S12d, Supporting Information) is specifically designed for singlet‐triplet leakage in asymmetric complexes as discussed in Knecht et al.^[^ ^63^ ^]^ Experimental details, such as the RF‐field locking power, irradiation frequency, loop counter, and delay time for exchange to free substrate form can be found in Experimental Section.

- ^15^N‐labelled ammonia

Performing SABRE at high field allows to fine‐tune the flow of magnetization toward ^15^N. In a system containing ^15^N‐labelled ammonia (dissolved in deuterated methanol via the ammonium buffer solution), a maximal enhancement in ^15^N of free ammonia surpassing three orders of magnitude (Figure 7a) was achieved. The high efficiency of hyperpolarization observed could originate from the fact that most of the ammonia species are ND_3_. Relaxation of ^15^N is therefore suppressed substantially in free form due to the absence of protons with a larger gyromagnetic ratio inducing relaxation. A *T*_1_ of 71.95 s was measured for the lifetime of ^15^N hyperpolarization in free ammonia by fitting an exponential decay onto a series of consecutive 2° low‐angle pulses separated by a 5 s repetition delay (Figure S10, Supporting Information). The main active catalyst configuration present is of course complex I (see Figure 4d) since no other co‐ligand is used.

Access to ^15^N hyperpolarized free ammonia is highly promising since this bio‐marker takes a central role in the nitrogen economy of plants as both a growth and nitrogen assimilation source.^[^ ^3^, ^79^, ^80^ ^]^ Experimental freedom to redistribute hyperpolarization inside the spectrometer to ^15^N ammonia using the SABRE‐SLIC approach can be used for monitoring reaction pathways. Besides circumventing interruptive field‐cycling activities, working solely at high‐field for hyperpolarization of ammonia in protic solvents provides an additional interesting opportunity. Since the exchangeable protons in ammonia are highly susceptible to protic solvent exchange, hyperpolarization at ultra‐low field µT regimes using p‐H_2_ and SABRE catalysts often results in fast redistribution of polarization to the huge number of protons and deuterons present in the system. Zeeman interactions generally distinguishing ^15^N, ^1^H, and ^2^D nuclei are simply negligible at µT fields generating altogether an efficient hyperpolarization sink.^[^ ^43^, ^81^ ^]^

- ^15^N‐labelled ammonia + alanine at ^15^N natural abundance

In a system containing ^15^N‐labelled ammonia in combination with alanine at natural abundances, the enhancement of hyperpolarized ^15^N of free ammonia dropped as noticed in Figure 7b. This decrease could be attributed to a potentially reduced ligand exchange rate in the mixed‐ligand complexes (complex II–VI Figure 4d) impacting both the refreshment rate of p‐H_2_ and the exchange of ammonia targets.

- ^15^N‐labelled alanine + ammonia at ^15^N natural abundance.

Hyperpolarization of free ^15^N‐alanine remained limited with the RF‐field locking approach (Figure 7c), yet it still suffices in enhancing ^15^N magnetization 12‐fold. Ammonia is hypothesized to create a favorable balance between strong competition for the association on the catalyst and promoting alanine to dissociate, allowing co(ligands) to exchange. Other co‐ligands that were employed (dimethylsulfoxide,^[^ ^57^ ^]^ acetonitrile,^[^ ^58^ ^]^ acetone,^[^ ^59^ ^]^ pyridine,^[^ ^54^ ^]^ and additionally triphenylphosphine,^[^ ^60^ ^]^ benzylamine and taurine) did not facilitate this favorable balance. For increasing hyperpolarization levels even further, optimization of the high‐field polarization transfer schemes in a system where faster exchange of AAs on iridium would be promoted is still necessary. The experimental control inherent to the all‐high‐field approach, however, invites to rapidly conduct this follow‐up research across the parahydrogen community, hopefully in parallel with field‐cycling techniques^[^ ^82^ ^]^ to boost hyperpolarization levels even further.

## 3 Conclusions

In this work, parahydrogen‐induced hyperpolarization of ^15^N in the amino acid alanine was accomplished reversibly without modifying the target amino acid. We adopted an all‐high‐field SABRE approach where both i) the hyperpolarization events and ii) the elucidation procedure could be mutually optimized at high‐field. First, detailed structure elucidation of the active spin transfer catalysts was provided, which subsequently led to hyperpolarization of both catalyst‐bound and free alanine in solution.

A critical step in these studies was to ensure monodentate ligation of the amino acid with the iridium spin transfer catalyst. Monodentate ligation of alanine on the SABRE active complexes was induced by introducing ammonium hydroxide buffer to deuterated methanol, which progressively produced ammonia as a strong competing ligand. The hydrated ammonium buffer not only enabled monodentate binding in the equatorial plane of the catalyst via the amine functionality of alanine, but the ammonium buffer addition also assisted in the structure elucidation of all catalyst complexes present in the solution. The detailed elucidation used ^1^H/D scrambling caused by the addition of protons to a deuterated solution enabled hydride fingerprinting (i.e., isotopological fingerprinting). 2D‐ZQ‐NMR gave us then access to the full structural information embedded in the isotopological fingerprints in case spectral overlap in 1D hydride spectra did not give sufficient resolution.

To achieve high‐field ^15^N hyperpolarization on free pristine alanine and ammonia, both SABRE‐INEPT and SABRE‐SLIC techniques were employed. Transfer of hydride spin polarization to ^15^N with SABRE‐INEPT was shown to be effective to polarize ligated ^15^N nuclei of alanine and ammonia. After the implementation of a variable exchange period, also an increase of ^15^N magnetization in free alanine and ammonia was observed. By monitoring and fitting the flow of magnetization from ligated to free alanine, the monodentate catalyst complexes were pinpointed to be most SABRE active.

Even though the increase in ^15^N polarization in free alanine remained modest (an order of magnitude) compared to free ammonia (three orders of magnitude) via the SABRE‐SLIC technique, this report has established the crucial first steps in the development of a general methodology optimized to maximize hyperpolarization in challenging SABRE targets. Unlocking hyperpolarization of other types of amino acids, such as lysine, glycine, histidine or others, utilizing varying temperatures,^[^ ^83^ ^]^ pH, solvent or even the co‐ligand is therefore expected sooner or later. Finally, we believe the high‐field method for hyperpolarizing ^15^N heteronuclei can be an excellent iteration toward alternative field‐cycling (SABRE‐SHEATH) techniques since many catalytic insights (stereochemistry and kinetics) remain mutually regulatory at these conditions despite working at ultra‐low magnetic fields.

## Conflict of Interest

The authors declare no conflict of interest.

## Author Contributions

E.B. and J.A.M. conceived and supervised the project. J.G.K. and J.M.T. provided technological and scientific support as part of a co‐development between Bruker Biospin and KU Leuven. E.V. performed the experimental work. All authors contributed to writing the manuscript. Late Francis Taulelle is acknowledged for his initial support of the amino acid hyperpolarization project.

## Supporting information

Supporting Information

## Data Availability

The data that support the findings of this study are openly available in Harvard Dataverse at https://doi.org/10.7910/DVN/A8TEEI, reference number 0.
