# Supplementary material for: Reversible Parahydrogen Induced Hyperpolarization of 15N in Unmodified Amino Acids Unraveled at High Magnetic Field
Source: Adv Sci (Weinh). 2023 May 21;10(23):2207112. doi: 10.1002/advs.202207112 (PMC10427394; doi:10.1002/advs.202207112)
Supplement: Supplementary file 1 — Supporting Information [file ADVS-10-2207112-s001.pdf]

ADVANCED SCIENCE
Open Access

Supporting Information

for *Adv. Sci.*, DOI 10.1002/advs.202207112

Reversible Parahydrogen Induced Hyperpolarization of ^{15}N in Unmodified Amino Acids Unraveled at High Magnetic Field

*Ewoud Vaneeckhaute, Jean-Max Tyburn, James G. Kempf, Johan A. Martens and Eric Breynaert**

Reversible NMR Hyperpolarization of ^{15}N in Unmodified Amino Acids Unravelled at High Magnetic Field

Ewoud Vaneeckhaute[a,b,c], Jean-Max Tyburn[d], James G. Kempf[e], Johan A. Martens[a,b,f] and Eric Breynaert*[a,b,f]

[a] COK-kat, Centre for Surface Chemistry and Catalysis – Characterization and Application Team, KULeuven, Celestijnenlaan 200F, box 2461, B-3001, Belgium.
[b] NMRCoRe, NMR/X-Ray platform for Convergence Research, KULeuven, Celestijnenlaan 200F, box 2461, B-3001 Leuven, Belgium.
[c] Univ Lyon, CNRS, ENS Lyon, UCBL, Université de Lyon, CRMN UMR 5280, 69100 Villeurbanne, France.
[d] Bruker Biospin, 34 Rue de l'Industrie BP 10002, 67166 Wissembourg Cedex, France.
[e] Bruker Biospin, 15 Fortune Dr., Billerica, 01821 Massachusetts, United States
[f] Deutsches Elektronen-Synchrotron DESY - Centre for Molecular Water Science (CMWS), Notkestraße 85, 22607 Hamburg, Germany

*Corresponding author (email): Eric.breynaert@kuleuven.be

Table of Figures

Figures	Description
Figure S1	Schematic representation of the in-house designed automated parahydrogen bubbling setup for hyperpolarization of heteronuclei at high magnetic field
Figure S2	Catalytic cycle of a reversible SABRE experiment to induce molecular hyperpolarization with p-H_2 on a target containing lone electron pairs
Figure S3	The 2D-ZQ-NMR experiment used for isotopological fingerprinting with product operator evolution description
Figure S4	The molar distribution of ^{1}H/D isotopologues of N-functionalities as function of the protonation degree of a deuterated solvent
Figure S5	$[Ir(IMes)(H_2)(COD)(S_{eq})]Cl^-$ intermediary complex after p-H_2 activation of IrCl(COD)IMes with(out) isotopic scrambling and with(out) ^{15}N-labelling of ammonia
Figure S6	2D-ZQ plots visualizing the isotopological variation of the N-functionality binding with the $[Ir(IMes)(H_2)(COD)(S_{eq})]Cl^-$ complex
Figure S7	Active $[Ir(IMes)(H_2)(S_{eq1}S_{eq2}S_{ax})]Cl^-$ SABRE complex with(out) isotopic scrambling and with(out) ^{15}N-labelling of ammonia and/or ^{15}N-labelling of alanine
Figure S8	Hydride regions of the isotopically fingerprinted $[Ir(IMes)(H_2)(S_{eq1}S_{eq2}S_{ax})]Cl^-$ SABRE complex in presence of alanine and ammonia at 298K and 278K
Figure S9	Selective ^{15}N decoupling correlation experiment using hyperpolarized hydrides
Figure S10	Longitudinal relaxation data of hyperpolarized ^{15}N magnetization in free ammonia
Figure S11	Structure of L-alanine based on 1D and 2D ^{1}H-NMR
Figure S12	NMR pulsetrains used for hyperpolarization and characterization of hydrides, and heteronuclei in ligated and free ligands

Experimental Methods

Synthetic procedures

^{1}H-labelling

22.5 mM of L-alanine (Sigma-Aldrich) (Figure S11) was dissolved in an alcohol-water mixture composed of 1 mL methanol-d_4 (99.9% deuteration, Sigma-Aldrich) and 50 µL NH_4OH (30 wt% NH_3 in H_2O, Sigma-Aldrich). Note that hydrated ammonia has a two-fold purpose here: (i) ammonia serves as coligand to influence the ligation state of L-alanine on the transition metal complex and (ii) the ^{1}H's present in the aqueous solution of ammonia also serves as ^{1}H-labelling agent to induce ^{1}H-D scrambling for catalyst structure determination. Finally, 0.78 mM IrCl(COD)(IMes) catalyst precursor (with IMes: 1,3-Bis(2,4,6-trimethylphenyl)imidazol-2-ylidene and COD: cyclooctadiene) was added to the mixture (Figure S2,I). The total molar ratio in the final solution between catalyst:ligand:coligand was 1:9.58:331. The square planar catalyst precursor was synthesized in Schlenk conditions according to the published procedure.[1] After sonification (5 minutes), a yellow alcohol-water sample was ready for use to become activated by p-H_2 in the high-field bubbling setup.

^{15}N-labelled hydrated ammonia

An identical hyperpolarization mixture as described above was also prepared but with ^{15}N-labelled hydrated ammonia. The addition of isotopically enriched ^{15}N ammonia helps in verifying the catalyst structure due to visible *J*-splitting of the trans-positioned hydrides across the abundant heteronuclei. This structural information supports the insights gathered from the ^{1}H-labelling technique combined with 2D-ZQ-NMR. Moreover, the same hyperpolarization mixture is also employed for high-field transfer of spin order from p-H_2 towards the ^{15}N nuclei of ammonia in ligated and free form using SABRE-INEPT and SABRE-SLIC experiments.

^{15}N-labelled L-alanine

Analogous to the case where ^{15}N-labelled hydrated ammonia is used to substantiate the catalyst structure, ^{15}N-labelled L-alanine is employed as a verification for monodentate ligation to support the ability for SABRE-derived signal enhancement at high-field conditions. Again, an identical mixture with the same molar contributions of catalyst precursor and coligand is prepared but now with ^{15}N-labelled L-alanine. Additionally, a mixture with minimal isotopological scrambling (minimal amount of ^{1}H's present) in combination with ^{15}N-labelled alanine was prepared by adding ND_3OD instead of NH_4OH as the ammonium buffer. The same molar ratios of ligand/co-ligand/v-catalyst were employed. High-field transfer of spin order from p-H_2 towards the ^{15}N nuclei of amino acids in ligated and free form using SABRE-INEPT and SABRE-SLIC experiments are available in the results and discussion.

Hyperpolarization hardware and NMR experiments

High-field bubbling setup

Activation of the pre-catalyst in the presence of L-alanine and coligand ammonia is performed by bubbling parahydrogen (p-H_2) through a liquid test sample at high magnetic field (800 MHz). 90% p-H_2 is supplied by a p-H_2 generator (Bruker, BPHG-90) which cryo-cools electrolytically produced hydrogen to 38K in the presence of a catalyst. The maximum outlet pressure is 6 bars with a volumetric flow rate of 0.2 L/min. 500 µL of the prepared hyperpolarization mixtures are inserted in a modified quartz 5 mm NMR tube which can be screwed gas-tight onto a PEEK reactor head with both in- and output lines for automated p-H_2 administration and gas evacuation respectively. Bubbling was performed using fluorinated ethylene propylene (FEP) 1/32'' microtubing stretching to the bottom of the NMR tube.

A detailed description of the used materials for designing the gas-tight NMR bubbling tube is presented in Figure S1. The PEEK reactor head together with the quartz 5mm tube is connected to a spectrometer-controlled, in-house designed bubbling setup. Based on the Bruker BPHP polarizer prototype lay-out[2], gas bubbling becomes back-pressure controlled (valve 3, Figure S1) up to 6 bars. Reproducibility is guaranteed by the TTL communication between the NEO console and the BPHP polarizer enabling pulse sequence control over bubbling duty cycles. A schematic representation of the full layout is displayed in Figure S1.

Bubbling cycles preceding NMR pulsetrains

Two separated bubbling phases are integrated to ensure efficient sensitivity-enhanced catalyst characterization using 1D hydride responses influenced by selective ^{15}N-labelling or 2D-ZQ hydride plots. After administration of each independent hyperpolarization mixture to the 5mm NMR tube, initial p-H_2 activation of the square planar IrCl(COD)(IMes) precursor (Figure S2,I) is facilitated at 298K by a 5-minute bubbling period at elevated pressure (5 bars). This generates an intermediate SABRE complex where chlorine is evacuated and cyclooctadiene is still ligated (Figure S2,II). Evacuation of chlorine and hydrogenation of cyclooctadiene opens new positions for three ligands (Figure S2,III); either occupied by L-alanine in monodentate or bidentate fashion or by coligand ammonia. After the conversion of the square planar to the octahedral configuration of the SABRE catalyst, shorter bubbling periods (ranging from 1 minute to 5 s) are implemented before each structure determination focused 1D or 2D-ZQ-NMR experiment or high-field hyperpolarization experiments. Unlike other reports[3,4], no inter-increment bubbling is used

to visualize the ZQ evolution of the isotopological inequivalent hydrides, since triplet magnetization remains present during the entire 2D experiments. This results in the fast observation of isotopological fingerprints under the 1-minute mark (not including the p-H_2 bubbling).

1D NMR for high-field observation of hyperpolarized hydrides

Spontaneous singlet-triplet conversion (Figure S3) in mixed-ligand SABRE complexes at high-field results in non-equilibrium longitudinal two-spin order embedded in the p-H_2 derived hydrides. In contrast to equilibrium Zeeman magnetization, longitudinal two-spin order can only be visualized using a $\pi/4$ pulse instead of the traditional $\pi/2$ pulse in the ^{1}H-channel. Each 1D NMR experiment probing the hydrides is therefore equipped with a $\pi/4$ pulse angle (Figure S12a). As a result, antiphase magnetization instead in-phase absorptive signals is produced and observed for hydrides being part of an asymmetric transition metal complex. Selective ^{15}N-decoupling in the case of ^{15}N-labelled substrates is applied as well (Figure 5b,d) to study its influence on the hydride splitting in ^{1}H. Selective decoupling is a complementary method used in the report to gain insight into the structural correlation between ^{15}N and ^{1}H resonances alike in the mixed-ligand complexes.

2D hyperpolarized Zero-Quantum NMR experiment

Isotopological fingerprinting using ^{1}H-labelling of the non-labelled (^{15}N) mixtures results in the spontaneous generation of observable sensitivity-enhanced triplet magnetization in hydrides facilitated by isotopic differentiation of cis/trans ligands with exchangeable protons. The non-equilibrium longitudinal two-spin order present in the triplet magnetization embeds ZQ coherences that evolve under the chemical shift difference (ZQ) or the chemical shift summation (DQ) of hydride partners. To extract these coherences (pulsetrain in Figure S3a,b), a simple $(\pi/2)_x$ pulse transforms the longitudinal triplet magnetization into a mixture of ZQ and DQ coherences (Figure S3b). Effective coherent evolution is then monitored under period t_1 (Figure S3c). Since our interest is focused on the ZQ terms, filtering ZQ from DQ is performed via a pulsed field homospoil gradient (5%, 3ms) (Figure S3d). The remaining magnetization, only ZQ in character at this point, is afterwards transformed into antiphase magnetization via a second broadband $(\pi/2)_x$ pulse (Figure S3e) and then acquired. A single bubbling period of 1 minute sufficed for enough triplet magnetization to overcome the entire ZQ evolution monitoring period. A total of 256 transients with 1 transient per increment was used for the construction of the ZQ plots in Figure 4a,b. The relaxation delay between each increment could be minimized to 100 ms because effective polarization is not from Zeeman origin, but the parahydrogen singlet state chemically transformed into triplet magnetization. All processing, phase and baseline corrections were performed using Mestrenova v12 from Mestrelab Research S.L.[5] Double Fourier transformation in absolute mode with Sine Bell adjustment and zero filling to 4096 (t_1) x 4096 (t_2) dimension constructed the 2D-plots in Figure 4a and b in the main manuscript.

SABRE-INEPT and adapted SABRE-INEPT for exchange profiling

For the construction of the hyperpolarized 1D ^{15}N plots in Figure 5, the SABRE-INEPT pulsetrain depicted in Figure S12c is used. Knowledge of the chemical shift of ligated ^{15}N-labelled ammonia and ^{15}N-labelled L-alanine is an important step towards the hyperpolarization of their free counterpart. The first $\pi/4$ pulse in the ^{1}H-channel is to excite the triplet magnetization of the hydrides. All other pulses both in ^{1}H and ^{15}N are designed to generate hydrides based $\hat{I}_x\hat{S}_z$ antiphase coherences ($\hat{I}$ correlates to a hydride, $\hat{S}$ to the ^{15}N nuclei ligated to the active metal centre) that evolve via *J*-coupling into increased antiphase magnetization $\hat{I}_z\hat{S}_x$ on the heteronuclear spin. An offset resonance of δ -22.4 ppm in ^{1}H and δ 0 ppm in ^{15}N was used to ensure equal excitement of all nuclear spins. Based on a $J_{15N\text{-}H}$ coupling of 15.75 Hz between the hydrides and ^{15}N nuclei across the transition metal, the refocusing delays τ_1 and τ_2 were chosen to be 60 ms and 30 ms respectively. Decoupling in the ^{1}H- and D-channel during acquisition clarifies the ^{15}N spectra even further. Bubbling was set to 1 min before the first pulse. Note that even though the ligands are already isotopically enriched with ^{15}N, without the transfer of polarization from p-H_2-derived hydrides, no visible NMR signal in ^{15}N can be witnessed. This makes SABRE-INEPT necessary for the elucidation of ^{15}N resonances belonging to ammonia or alanine. In the adapted version, two additional $\pi/4$ pulses on ^{15}N were included with a variable exchange time (t_e) in between them. This allowed for monitoring the exchange of ligands on and off the metal centre. Finally, ^{15}N chemical shift information is rather crucial for selectively irradiating the correct resonances to induce polarization transfer to free substrates using SABRE-SLIC.

SABRE-SLIC

The SABRE-SLIC pulsetrain presented in Figure S12d was deduced from Knecht *et al.*[6] to enable both singlet and triplet magnetization as a source of spin order for polarization transfer. The hyperpolarization pulsetrain makes use of RF spin-locking on ^{15}N nuclei attached to the catalyst to induce ^{15}N hyperpolarization of free substrates. The correct condition for spin mixing and population exchange happening at level anticrossings are mimicked this way to also occur at high field instead of the required ultra-low fields of a few µT.

The RF-driven SABRE pulsetrains were originally designed by Theis and co-workers to only rely on singlet magnetization for fuelling polarization in free heteronuclei.[7] However, since singlet-triplet mixing is apparent in our mixtures via coligand ammonia influencing the asymmetry of the complex, the adapted version presented here implements an additional $\pi/2$ pulse in the ^{1}H-

channel and off-resonant ^{15}N field-locking to generate hyperpolarized $\hat{S}_z$ magnetization in ^{15}N directly. For observation of enhanced ^{15}N magnetization in free ammonia, irradiation was set to δ -41 ppm with an amplitude of 40 dB and a duration of 100 ms. For alanine, the irradiation was positioned at δ -5.5 ppm, also with an amplitude of 40 dB and duration of 200 ms. The chemical shifts were derived from the SABRE-INEPT experiments that specifically target hyperpolarization of the monodentate ligated forms of the alanine and ammonia substrates. Bubbling was set to only 1 second since short but repetitive cycles of the RF-field locking during bubbling are beneficial for increased polarization enhancements (analogous to multiple contact cross polarization). A repetition of 50 cycles was enough to experience ^{15}N enhancement in free ammonia and alanine. The time in between each cycle (τ_d) was set to 1 s to ensure polarization established in the free form of the substrates cannot be destroyed. The value of τ_d is dependent on the exchange rate of p-H_2 and (co)ligands onto the active metal centre.

High-field bubbling setup

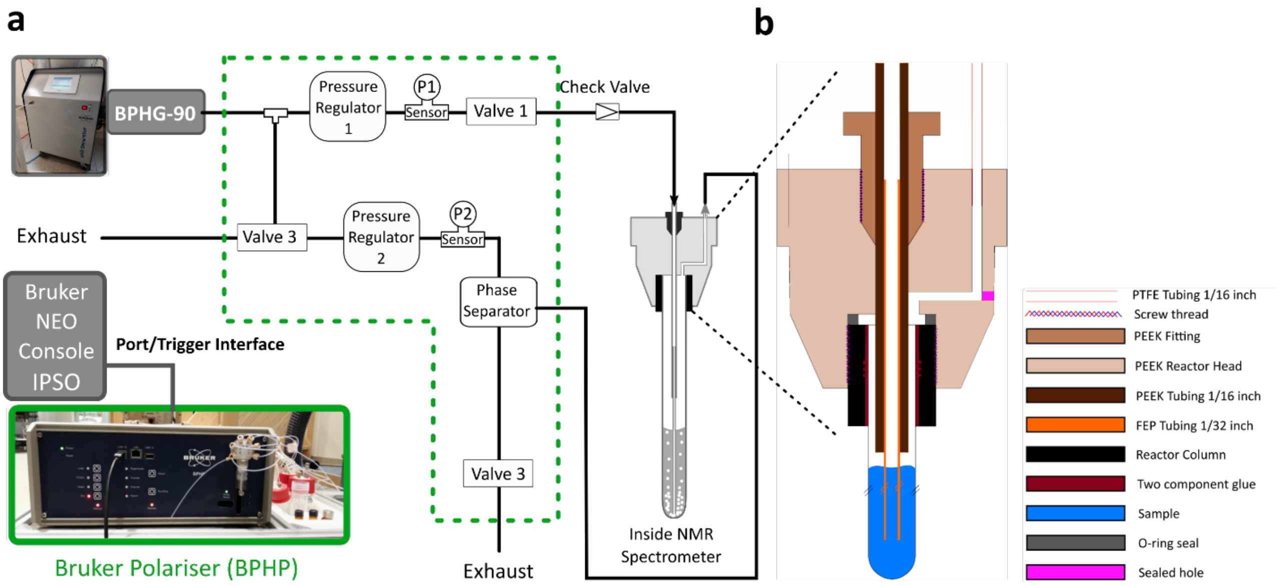

Figure S1. In-house design for automated pressure-controlled parahydrogen bubbling through a catalyst solution situated in a 5 mm quartz NMR tube at high field. a) Schematic layout to enable automated gas administration. For p-H_2 supply, the external Bruker BPHG-90 generator provides a continuous source of 90% p-H_2. The Bruker BPHP polarizer prototype handles automated valve and pressure control in connection with the spectrometer (TTL) and PC (USB). b) In-house designed PEEK reactor head combined with a 5 mm quartz NMR tube for gas-tight administration and evacuation of parahydrogen for elucidation of uncharacterized catalyst mixtures. Captions for the different materials used for the construction of the bubbling device are included.

Results and Discussions

Catalytic cycle of a reversible SABRE experiment using p-H_2 and IrCl(COD)IMes

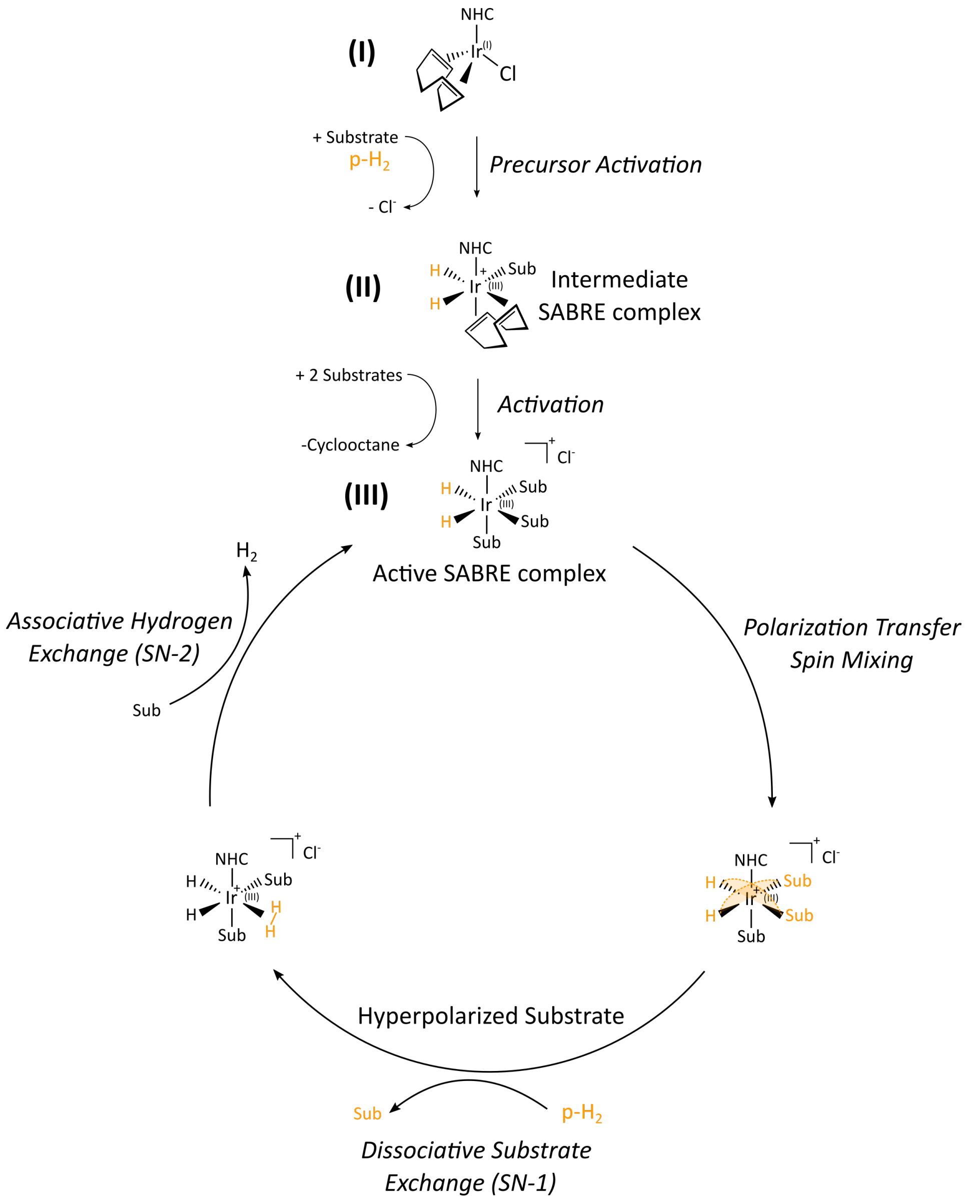

Figure S2. Catalytic cycle of a reversible SABRE experiment to induce molecular hyperpolarization with p-H_2 on a target containing lone electron pairs. In this case amino acids are the targets. Activation of the tetrahedral N-Heterocyclic Carbene (NHC) precursor catalyst IrCl(COD)IMes (I) is necessary to generate first the intermediate [Ir(IMes)(H_2)(COD)(S_{eq})]Cl^- complex (II) and then the active octahedral [Ir(IMes)(H_2)($S_{eq1}S_{eq2}S_{ax}$)]Cl^- complex (III).

2D zero-quantum NMR experiment

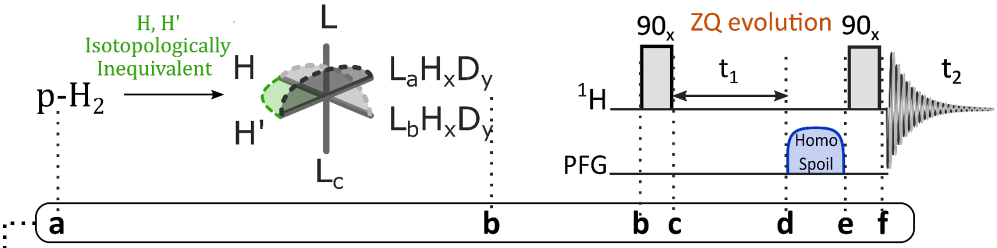

Singlet-Triplet Mixing

a $\hat{\rho}_S = \frac{\hat{1}}{4} - \frac{1}{2}(2\,\hat{I}_{1z}\hat{I}_{2z} + 2\hat{I}_{1x}\hat{I}_{2x} + 2\hat{I}_{1y}\hat{I}_{2y}) \longrightarrow \hat{\rho}_{T0} = \frac{\hat{1}}{4} - \frac{1}{2}(2\,\hat{I}_{1z}\hat{I}_{2z})$

$ZQC = \hat{I}_{1x}\hat{I}_{2x} + \hat{I}_{1y}\hat{I}_{2y}$

Dephases/Decays

b $\hat{I}_{1z}\hat{I}_{2z} \xrightarrow{(\pi/2)\hat{I}_{1x} + (\pi/2)\hat{I}_{2x}} \hat{I}_{1y}\hat{I}_{2y}$ ZQC/DQC Mixture

c $\hat{I}_{1y}\hat{I}_{2y} \xrightarrow{\Omega_1 t\hat{I}_{1z} + \Omega_2 t\hat{I}_{2z}} \{cos(\Omega_1 t_1)\hat{I}_{1y} - sin(\Omega_1 t_1)\hat{I}_{1x}\}\{cos(\Omega_2 t_1)\hat{I}_{2y} - sin(\Omega_2 t_1)\hat{I}_{2x}\}$

d

$$\begin{aligned}
&\hat{I}_{1x}\hat{I}_{2x}\{1/2\,cos[t_1(\Omega_1 - \Omega_2)] - 1/2\,cos[t_1(\Omega_1 + \Omega_2)]\}\\
&+\hat{I}_{1y}\hat{I}_{2y}\{1/2\,cos[t_1(\Omega_1 - \Omega_2)] + 1/2\,cos[t_1(\Omega_1 + \Omega_2)]\}\\
&+\hat{I}_{1y}\hat{I}_{2x}\{1/2\,sin[t_1(\Omega_1 - \Omega_2)] - 1/2\,sin[t_1(\Omega_1 + \Omega_2)]\}\\
&-\hat{I}_{1x}\hat{I}_{2y}\{1/2\,sin[t_1(\Omega_1 - \Omega_2)] + 1/2\,sin[t_1(\Omega_1 + \Omega_2)]\}
\end{aligned}$$

$\xrightarrow{\text{Homospoil ZQfilter}}$

$$\begin{aligned}
&1/2\hat{I}_{1x}\hat{I}_{2x}\,cos[t_1(\Omega_1 - \Omega_2)] + 1/2\hat{I}_{1y}\hat{I}_{2y}\,cos[t_1(\Omega_1 - \Omega_2)]\\
&+1/2\hat{I}_{1y}\hat{I}_{2x}\,sin[t_1(\Omega_1 - \Omega_2)] - 1/2\hat{I}_{1x}\hat{I}_{2y}\,sin[t_1(\Omega_1 - \Omega_2)]
\end{aligned}$$

$\|$

$$\begin{aligned}
&1/2ZQC_x\,cos[t_1(\Omega_1 - \Omega_2)]\\
&+1/2ZQC_y\,sin[t_1(\Omega_1 - \Omega_2)]
\end{aligned}$$

e

$$\begin{aligned}
&1/2ZQC_x\,cos[t_1(\Omega_1 - \Omega_2)]\\
&+1/2ZQC_y\,sin[t_1(\Omega_1 - \Omega_2)]
\end{aligned} \xrightarrow{(\pi/2)\hat{I}_{1x} + (\pi/2)\hat{I}_{2x}}$$

f $+1/2\hat{I}_{1z}\hat{I}_{2x}\,sin[t_1(\Omega_1 - \Omega_2)] - 1/2\hat{I}_{1z}\hat{I}_{2y}\,sin[t_1(\Omega_1 - \Omega_2)]$

$\|$

$1/2(\hat{I}_{1z}\hat{I}_{2x} - \hat{I}_{1z}\hat{I}_{2y})\,sin[t_1(\Omega_1 - \Omega_2)]$

Figure S3. 2D-ZQ-NMR experiment used for isotopological fingerprinting. Product operator evolution is described from initial singlet-triplet mixing occurring at high field towards the effective manipulation of triplet magnetization using the 2D-ZQ pulsetrain displayed in the right top corner. Overall, steps **a-e** describe the transformation of magnetization over time under both processes (singlet-triplet mixing and 2D-ZQ-NMR) and are disclosed in detail.

Isotopological distribution of N-functionalities with exchangeable protons/deuterons

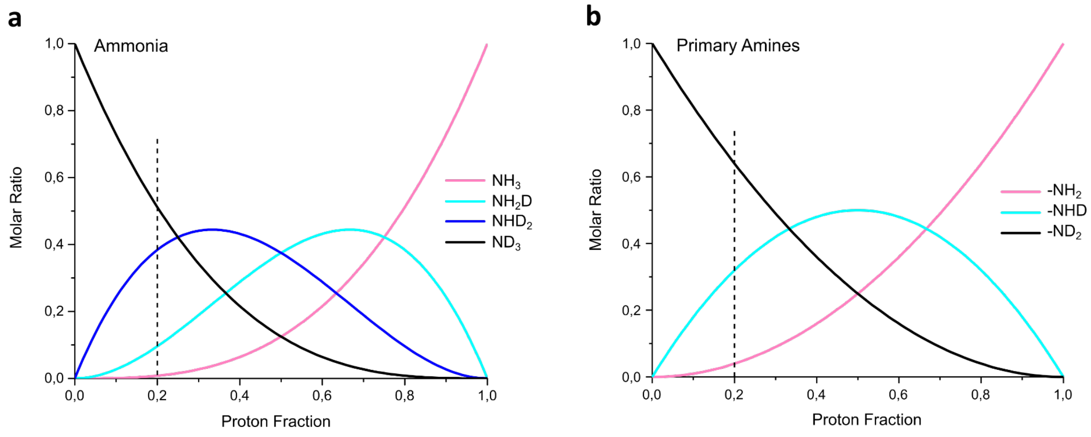

Figure S4. The molar distribution of ^{1}H/D isotopologues in function of the protonation degree of a deuterated solvent based on the binomial distribution of equation 1 from the main manuscript.[8] a) Since ammonia has a total of four isotopologues when ligated to the Ir-centre, at the current protonation degree of the samples under investigation (around 20.07 %), in practice only three will be visible in the ZQ plots (NH_3 is excluded). b) The primary amine functionality of the backbone of amino acids only can occur in three different isotopologues. At the same protonation degree of 20.07 %, in practice only two will be visible ($-NH_2$ is excluded).

Intermediary complex after p-H_2 activation of IrCl(COD)IMes with(out) isotopic scrambling and with(out) ^{15}N labelling of ammonia

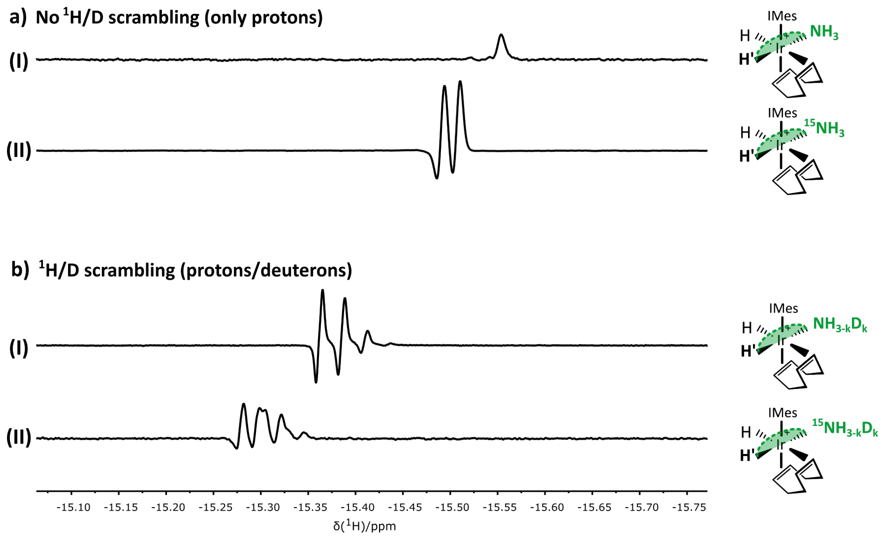

Figure S5. Hydride region after a $\pi/4$ pulse showing the intermediary SABRE complex $[Ir(IMes)(H_2)(COD)(S_{eq})]Cl^-$ after initial activation of the IrCl(COD)IMes precursor with p-H_2, alanine and hydrated ammonia. The trans-positioned hydrides with respect to ammonia in $[Ir(IMes)(H_2)(COD)(S_{eq})]Cl^-$ are shown since the effect of isotopologue chemical shift variation is more visible than the cis-positioned alternative. In a) non-deuterated methanol was used as a solvent to minimize proton/deuteron scrambling in combination with (I) natural abundant hydrated ammonia and (II) ^{15}N-labelled hydrated ammonia. In b) deuterated methanol was used in combination with (I) natural abundant hydrated ammonia and (II) ^{15}N-labelled hydrated ammonia.

2D zero quantum plot of intermediary complex after p-H_2 activation of IrCl(COD)IMes

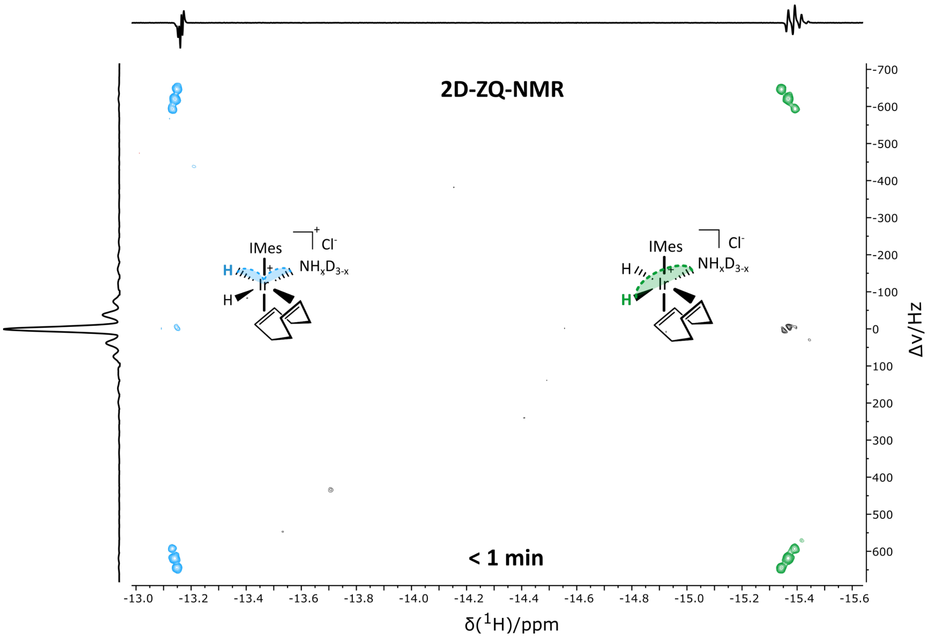

Figure S6. 2D-ZQ plots visualizing the isotopological variation of the N-functionality binding with the [Ir(IMes)(H_2)(COD)(S_{eq})]Cl^- complex. In this case, ammonia is associated since four isotopologues are present in the 1D projection as well as in the 2D ZQ plot (right below).

Active SABRE complex with(out) isotopic scrambling and with(out) ^{15}N labelling of ammonia and/or ^{15}N labelling of alanine

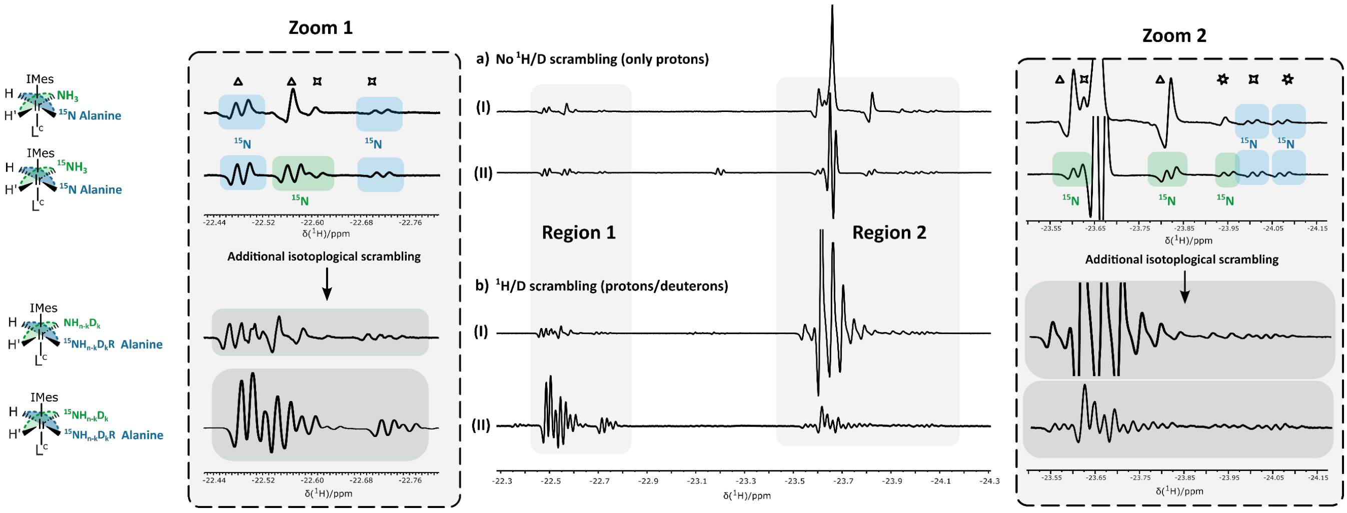

Figure S7. a) Hydride region after a $\pi/4$ pulse showing the asymmetric antiphase hydride couples in several active SABRE complexes with the general formula [Ir(IMes)(H_2)($S_{eq1}S_{eq2}S_{ax}$)]Cl^- where S_{eq1}, S_{eq2}, and S_{ax} are either ammonia or alanine. Region 1 shows the fingerprints of complexes with axial ligands (L_c) linking through an oxygen atom. Region 2 shows fingerprints of complexes with a nitrogen-containing functional group in their axial position (L_c), in this case, ammonia or amine from alanine. In a) non-deuterated methanol was used as a solvent to minimize proton/deuteron scrambling in combination with (I) natural abundant hydrated ammonia and ^{15}N-labelled alanine and (II) ^{15}N-labelled hydrated ammonia and ^{15}N-labelled alanine. The trans-J-coupling between the hydrides and ^{15}N nuclei of either ^{15}N-labelled ammonia and/or ^{15}N-labelled alanine reveals the association behaviour of both ligands once again. In b) deuterated methanol was used as a solvent in combination with (I) natural abundant hydrated ammonia and ^{15}N-labelled alanine and (II) ^{15}N-labelled hydrated ammonia and ^{15}N-labelled alanine. The zoomed regions clarify the hydride couples (two antiphase hydrides with the same symbol) and highlight the hydrides trans to alanine (blue) and ammonia (green).

Temperature dependency of the octahedral configuration of active SABRE catalyst in contact with ammonia and alanine

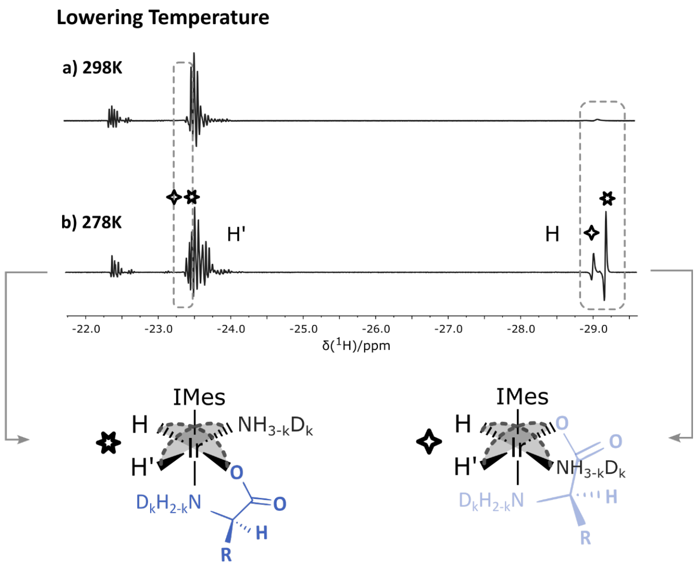

Figure S8. Temperature-dependent variation of the hydride region after a $\pi/4$ pulse showing the asymmetric antiphase hydride couples of several isotopological scrambled SABRE complexes with the general formula $[Ir(IMes)(H_2)(S_{eq1}S_{eq2}S_{ax})]Cl^-$ where S_{eq1}, S_{eq2}, and S_{ax} are either ammonia or alanine. a) Hydride region of the isotopically fingerprinted mixture of alanine and ammonia in the presence of an Ir-catalyst at 298K. b) Hydride regions of the isotopically fingerprinted mixture of alanine and ammonia in the presence of an Ir-catalyst at 278K. The hydride resonance at -29 ppm is originating from the oxygen of alanine binding equatorially in a bidentate fashion. This chelation effect must be avoided in case reversible exchange is required for hyperpolarization via SABRE. The corresponding SABRE inactive complexes are shown below. In case of bidentate ligation with oxygen equatorially positioned, two different diastereomers are possible.[9] This is the reason why two distinct hydride peaks trans to oxygen at -29 ppm arise, not to be mistaken with isotopological hydride scrambling in case N-functional groups are ligating.

Selective ^{15}N decoupling correlation experiment

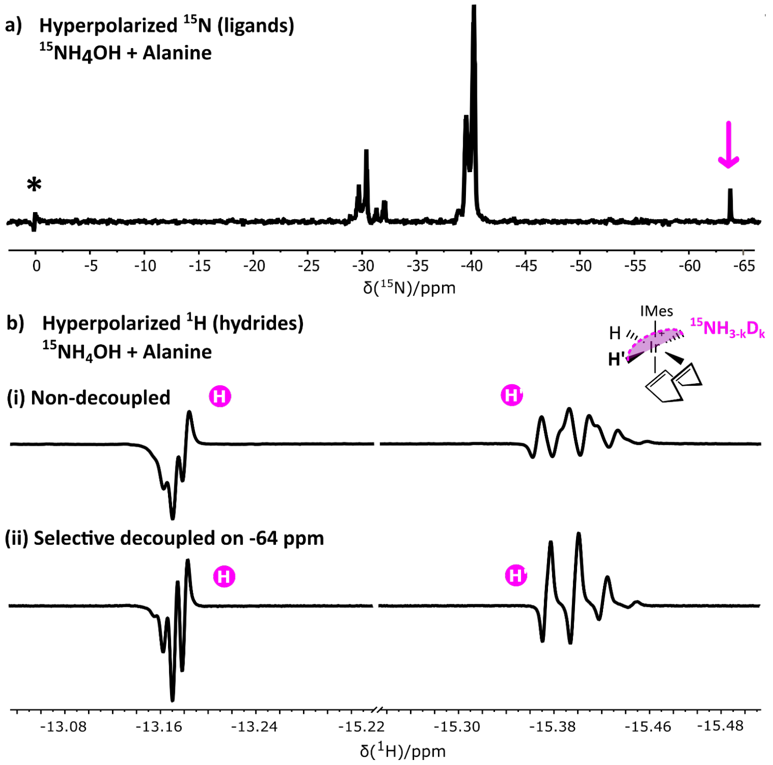

Figure S9. Selective ^{15}N decoupling correlation experiment when observing the hyperpolarized hydride region after p-H_2 bubbling and a $\pi/4$ pulse. a) Hyperpolarized ^{15}N resonances arising from ligated ^{15}N-labelled ammonia in competition with unlabelled alanine. Hyperpolarization is transferred from p-H_2 to the substrates via a J-based INEPT polarization transfer scheme (SABRE-INEPT) found in the experimental section of the main manuscript. b) ^{15}N resonances of ligated ammonia are related to the hydride resonances (i) based on selective ^{15}N decoupling (ii). When decoupling at -64 ppm, the hydride region showing the asymmetric antiphase hydride couples corresponding to $[Ir(IMes)(H_2)(COD)(NH_{3-k}D_k)]Cl^-$ is influenced.

Longitudinal relaxation of ^{15}N in free hyperpolarized ammonia

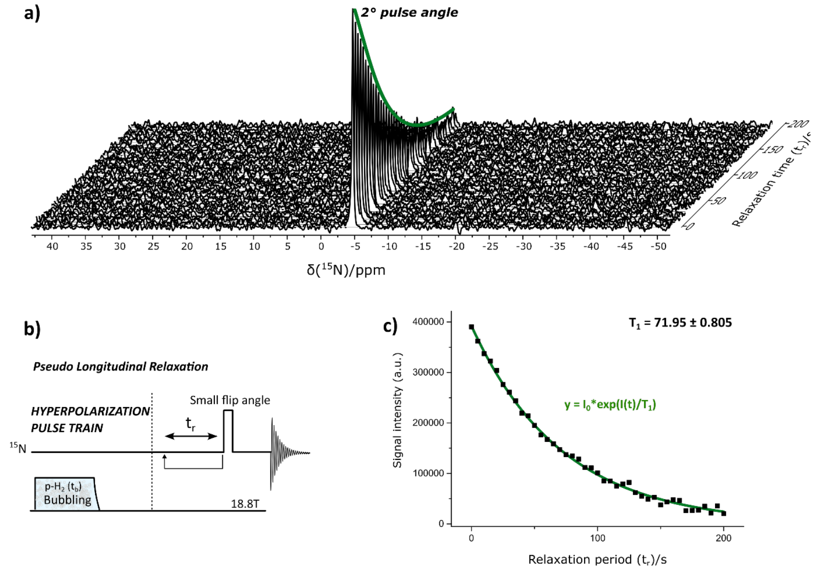

Figure S10. Longitudinal relaxation data of hyperpolarized ^{15}N magnetization in free ammonia. a) ^{15}N hyperpolarization decay after a series of consecutive 2° low angle pulses preceded by SLIC hyperpolarization transfer at high-field across a complex with p-H_2 accompanied by three ^{15}N-labelled ammonia ligands. The complex was prepared by dissolving 50 µL ^{15}N-labelled ammonium hydroxide in 1 mL deuterated methanol together with the Ir-precursor. b) Pulsetrain with a repetition period (t_r) of 5 s to monitor the hyperpolarization decrease over time. c) A T_1 of 71.95 s was fitted to the exponential decay of the hyperpolarized ^{15}N magnetization.

Structure of L-alanine derived from 1D ^1H-NMR and 2D ^1H-^1H COSY

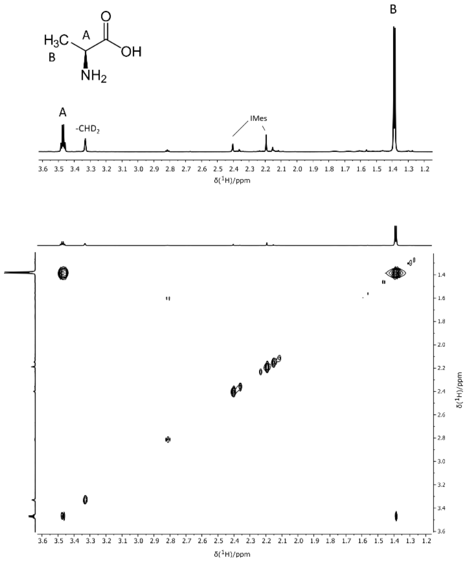

Figure S11. Structure of L-alanine used in the high-field SABRE hyperpolarization experiments. The 2D NMR correlates the A and B protons of through J-coupling.

Pulse sequences involving parahydrogen bubbling used in the manuscript

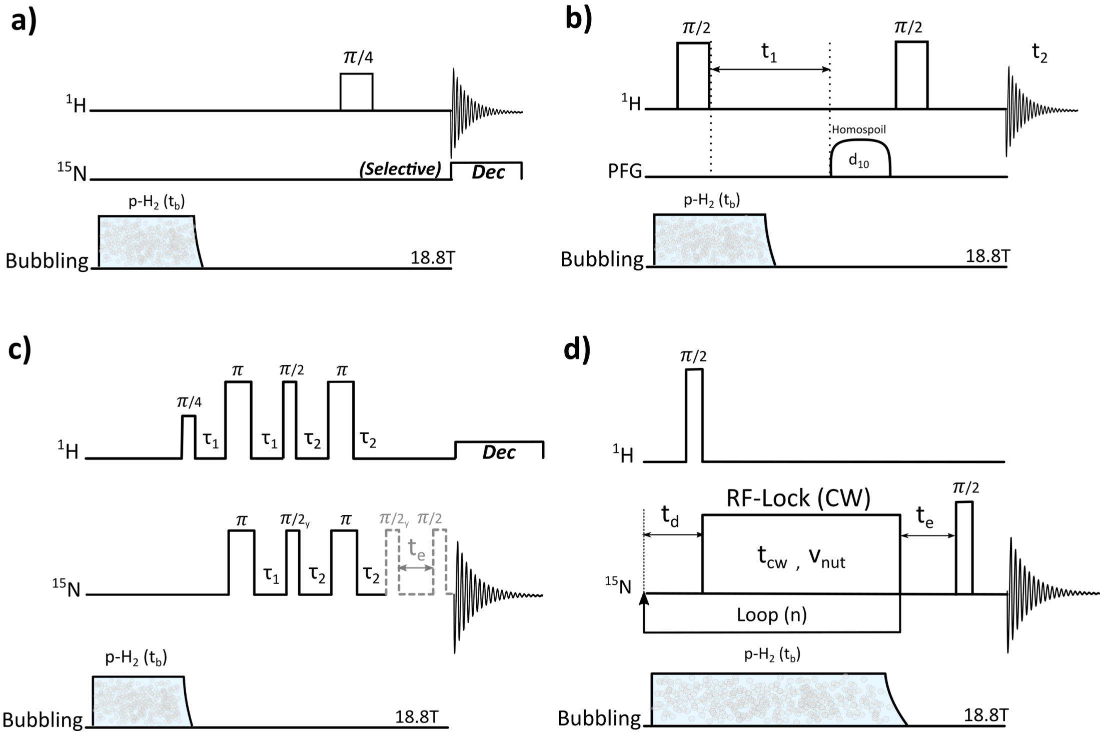

Figure S12. a) 1D NMR pulsetrain for probing non-equilibrium two-spin order in chemically inequivalent hydrides. In contrast to equilibrium Zeeman magnetization, longitudinal two-spin order can only be visualized using a $\pi/4$ pulse instead of the traditional $\pi/2$ pulse in the ^{1}H-channel. Selective decoupling in ^{15}N using CW irradiation is applied when ^{15}N-labelled substrates are used. b) 2D-zero-quantum NMR pulse sequence filtering zero-quantum evolution of isotopological inequivalent hydride couples with a rate proportional to their chemical shift difference. c) Pulsetrain for polarization transfer at high field of non-equilibrium triplet magnetization in hydrides to ^{15}N heteronuclei in ligated substrates using INEPT. The first $\pi/4$ pulse is again vital for the transformation of triplet magnetization in useable antiphase coherence to induce population exchange and sensitivity enhancement of ^{15}N. An adapted version of SABRE-INEPT including two additional $\pi/4$ pulses on ^{15}N with a variable exchange time (t_e) in between was employed for monitoring the exchange of ligands and inducing hyperpolarization in free substrates. d) Pulsetrain for high-field polarization transfer to ^{15}N heteronuclei in free substrates using an RF-field lock by continuous wave (CW) irradiation at the frequency of the heteronucleus in a ligated state. The bubbling is performed at high field during the RF-field lock and can be repeated multiple times before read-out. An exchange time in between each cycle (τ_d) ensures that hyperpolarization established in the free form of the substrates remains there. The value of τ_d is dependent on the exchange rate of p-H_2 and (co)ligands onto the active metal centre.

References

1. O. Torres, M. Martín and E. Sola, *Organometallics*, 2009, **28**, 863–870.
2. E. Vaneeckhaute, S. De Ridder, J. M. Tyburn, J. G. Kempf, F. Taulelle, J. A. Martens and E. Breynaert, *ChemPhysChem*, 2021, **22**, 1170–1177.
3. L. Sellies, I. Reile, R. L. E. G. Aspers, M. C. Feiters, F. P. J. T. Rutjes and M. Tessari, *Chem. Commun.*, 2019, **55**, 7235–7238.
4. N. K. J. Hermkens, M. C. Feiters, F. P. J. T. Rutjes, S. S. Wijmenga and M. Tessari, *J. Magn. Reson.*, 2017, **276**, 122–127.
5. M. R. Willcott, *J. Am. Chem. Soc.*, 2009, **131**, 13180–13180.
6. S. Knecht, A. S. Kiryutin, A. V. Yurkovskaya and K. L. Ivanov, *Mol. Phys.*, 2019, **117**, 2762–2771.
7. T. Theis, M. Truong, A. M. Coffey, E. Y. Chekmenev and W. S. Warren, *J. Magn. Reson.*, 2014, **248**, 23–26.
8. D. A. Barskiy, J. W. Blanchard, M. Reh, T. Sjoelander, A. Pines and D. Budker, *arXiv*.
9. A. N. Pravdivtsev, G. Buntkowsky, S. B. Duckett, I. V. Koptyug and J.-B. Hövener, *Angew. Chem. Int. Ed.*, 2021, **60**, 2–14.
